# Amide-to-Triazole Switch in Somatostatin-14-Based Radioligands: Impact on Receptor Affinity and In Vivo Stability

**DOI:** 10.3390/pharmaceutics16030392

**Published:** 2024-03-13

**Authors:** Xabier Guarrochena, Panagiotis Kanellopoulos, Anna Stingeder, Lisa-Maria Rečnik, Irene V. J. Feiner, Marie Brandt, Wolfgang Kandioller, Theodosia Maina, Berthold A. Nock, Thomas L. Mindt

**Affiliations:** 1 Institute of Inorganic Chemistry, Faculty of Chemistry, University of Vienna, Josef-Holaubek-Platz 2, 1090 Vienna, Austria; 2 Vienna Doctoral School in Chemistry, University of Vienna, Währinger Straße 42, 1090 Vienna, Austria; 3 Ludwig Boltzmann Institute Applied Diagnostics, AKH Wien c/o Sekretariat Nuklearmedizin, Währinger Gürtel 18-20, 1090 Vienna, Austria; 4 Department of Biomedical Imaging and Image Guided Therapy, Division of Nuclear Medicine, Medical University of Vienna, Währinger Gürtel 18-20, 1090 Vienna, Austria; 5 Molecular Radiopharmacy, INRaSTES, NCSR “Demokritos”, 15341 Athens, Greece; 6 Institute of Inorganic Chemistry, Faculty of Chemistry, University of Vienna, Währinger Str. 42, 1090 Vienna, Austria; 7 Joint Applied Medicinal Radiochemistry Facility, University of Vienna and Medical University of Vienna, 1090 Vienna, Austria

**Keywords:** somatostatin, peptide-based radiotracer, pansomatostatin, bioisosteres, amide-to-triazole substitution, 1,4-disubstituted 1,2,3-triazole, triazolo-peptidomimetic, metabolic stability, neprilysin, Entresto^®^

## Abstract

The use of metabolically stabilized, radiolabeled somatostatin (SST) analogs ([^68^Ga]Ga/[^177^Lu]Lu-DOTA-TATE/TOC/NOC) is well established in nuclear medicine. Despite the pivotal role of these radioligands in the diagnosis and therapy of neuroendocrine tumors (NETs), their inability to interact with all five somatostatin receptors (SST_1–5_R) limits their clinical potential. [^111^In]In-AT2S is a radiolabeled DOTA-conjugate derived from the parent peptide SST-14 that exhibits high binding affinity to all SSTR subtypes, but its poor metabolic stability represents a serious disadvantage for clinical use. In order to address this issue, we have replaced strategic *trans*-amide bonds of [^111^In]In-AT2S with metabolically stable 1,4-disubstituted 1,2,3-triazole bioisosteres. From the five cyclic triazolo-peptidomimetics investigated, only [^111^In]In-XG1 combined a preserved nanomolar affinity for the SST_1,2,3,5_R subtypes in vitro and an improved stability in vivo (up to 17% of intact peptide 5 min postinjection (pi) versus 6% for [^111^In]In-AT2S). The involvement of neprilysin (NEP) in the metabolism of [^111^In]In-XG1 was confirmed by coadministration of Entresto^®^, a registered antihypertensive drug, in vivo releasing the selective and potent NEP-inhibitor sacubitrilat. A pilot SPECT/CT imaging study conducted in mice bearing hSST_2_R-positive xenografts failed to visualize the xenografts due to the pronounced kidney uptake (>200% injected activity (IA)/g at 4 h pi), likely the result of the formation of cationic metabolites. To corroborate the imaging data, the tumors and the kidneys were excised and analyzed with a *γ*-counter. Even if receptor-specific tumor uptake for [^111^In]In-XG1 could be confirmed (1.61% IA/g), further optimization is required to improve its pharmacokinetic properties for radiotracer development.

## 1. Introduction

Since the discovery of somatostatin-14 (SST-14) by Guillemin et al. in 1972, this cyclic neuropeptide has been the focus of thorough investigations revealing its paramount relevance in medicine [1]. SST-14 exerts inhibitory and antisecretory actions by interacting with a group of G-protein-coupled receptors (GPCRs) comprising five subtypes (somatostatin receptor subtype 1–5, SST_1-5_R) [2]. The SST_1-5_R are expressed in various tissues and organs serving diverse regulatory functions, but they are also of significant importance in oncology by virtue of their high-density expression in different tumors [3,4]. Thus, neuroendocrine tumors (NETs), such as growth hormone-secreting pituitary adenoma, predominantly express SST_2_R, but concomitant expression of different subtypes is a common finding, as for example, in breast cancer [5]. Furthermore, human tumors devoid of SST_2_R expression may express one or more of other SSTR subtypes. For example, prostate cancer lesions predominantly express the SST_1_R [6,7].

**Figure 1:**
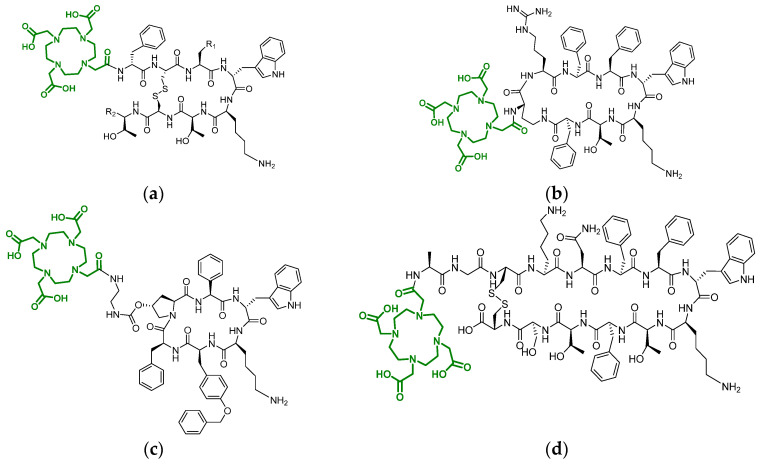
Structures of: (**a**) DOTA-TOC (R_1_ = 4-hydroxyphenyl/R_2_ = CH_2_OH), DOTA-TATE (R_1_ = 4-hydroxyphenyl/R_2_ = COOH) and DOTA-NOC (R_1_ = 1-naphthyl/R_2_ = CH_2_OH); (**b**), DOTA-KE108, (**c**) DOTA-SOM230; (**d**) AT2S. The DOTA chelator is indicated in a green color.

The clinical importance of SST_1-5_R biomarkers has been explored in nuclear medicine by the development of radiometal-labeled conjugates of cyclic octapeptides (TATE/TOC/NOC) with the universal macrocyclic chelator 1,4,7,10-tetraazacyclododecane-1,4,7,10-tetraacetic acid (DOTA) [8,9]. These truncated versions of SST-14 are SST_2_R-prefering and present higher in vivo stability as compared to the native peptide, resulting in improved pharmacokinetic properties [10]. DOTA-TATE/TOC labeled with either diagnostic (gallium-68, indium-111) or therapeutic radionuclides (lutetium-177, yttrium-90) have been successfully used in the clinic for the management of NETs, due to their high affinity toward the SST_2_R [11]. DOTA-NOC (Figure 1a) presents the widest affinity profile and is able to interact, in addition to the SST_2_R, with moderate (SST_3_R) to high affinity (SST_5_R) with other somatostatin receptor subtypes [9]. While some of the abovementioned radioligands (LUTATHERA^®^, NETSPOT^®^) have received approval by regulatory agencies for the imaging and treatment of NETs, they are still SST_2_R-preferring, a feature that limits their wider clinical indications [10].

**Figure 2:**
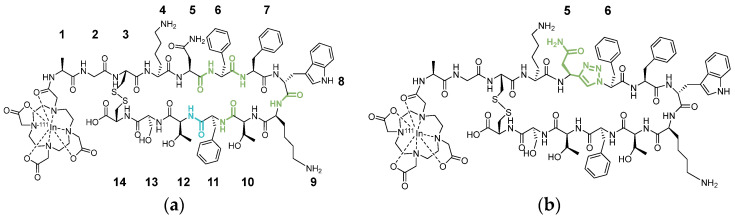
Structures of: (**a**) the reference compound [^111^In]In-AT2S; (**b**) the triazolo-peptidomimetic [^111^In]In-XG1 (**b**). The bonds highlighted in colors were subjected to the amide-to-triazole substitution (Table 1). The olive-green *trans*-amide bonds correspond to the reported positions cleaved by NEP and the turquoise position is an adjacent bond to a cleavage site.

Several attempts have been made to develop radiolabeled somatostatin analogs with high affinity toward all five SST_1-5_R, referred to as pansomatostatin-like [12]. SOM230 and KE180 are two multi/pan-somatostatin receptor ligands (SST_1-3, 5_R-/SST_1-5_R-affine, respectively) used for the development of clinically tested radioligands. Interestingly, a radiolabeled version of DOTA-KE108 (KE88, Figure 1b) displayed poor retention in SST_2_R-expressing tumors, limiting its clinical prospects [13]. Furthermore, the DOTA-conjugate of SOM230 (PA1, Figure 1c) was characterized by a high liver uptake, complicating the identification of lesions in the liver [14]. [^111^In]In-AT2S (Figure 1d) is a radiolabeled pansomatostatin-like peptide based on the SST-14 scaffold following two structural modifications [15]. First, the L-Trp^8^ is exchanged with d-Trp^8^, a substitution reported to increase the affinity for SST_2_R and metabolic stability in vivo [16]. Second, the Ala^1^ residue on the *N-*terminus is coupled to DOTA, enabling the radiolabeling with different metallic radionuclides and serving as N-terminal capping against exopeptidases at the same time [15]. Despite its pansomatostatin affinity profile, [^111^In]In-AT2S showed disadvantageously poor metabolic stability in blood circulation. It should be noted that neutral endopeptidase (NEP) has been involved in the fast breakdown of SST-14 and its analogs cleaving the Asn^5^-Phe^6^, Phe^6^-Phe^7^, Trp^8^-Lys^9^ and Thr^10^-Phe^11^ bonds of the 12mer cycle, as shown in Figure 2 [17,18,19,20].

Different approaches have been studied to address the fast degradation of SST-14 and its analogs, such as key amino acid substitutions, reduction of ring size or use of NEP-inhibitors, but results have been suboptimal thus far [15,18]. An alternative and yet unexplored approach in the case of SST-14 is the use of metabolically stable bioisosteres of amide bonds, which often represents an elegant strategy to improve the metabolic stability of peptides while preserving their biological function [21]. Among the many mimics of amide bonds reported (e.g., sulfonamides, semicarbazides, etc.), triazole heterocycles have emerged as reliable amide bond surrogates that can be incorporated efficiently into the backbone of peptides by convenient manual or automated solid-phase peptide synthesis (SPPS), utilizing the Cu(I)-catalyzed azide-alkyne cycloaddition (CuAAC) [22,23,24]. Metabolically, stable 1,4-disubstituted 1,2,3-triazoles (Tz) effectively mimic *trans*-amide nbonds whereas the 1,5-disubstituted regioisomers can serve as surrogates of *cis*-amide bonds [25]. The amide-to-triazole substitution strategy has previously been applied to different tumor-targeting linear peptides with high affinity toward their respective targets [26,27]. The obtained triazolo-peptidomimetics exhibited enhanced metabolic stabilities and maintained receptor affinities resulting in an increased tumor uptake in vivo (mice). In some cases, the receptor subtype selectivity of the peptide could be regulated or the affinity toward its target improved [28]. We now report the first application of this methodology to a cyclic tumor-targeting peptide, [^111^In]In-AT2S. Selected amide bonds in the cyclic part of SST-14, prone to cleavage by NEP (Asn^5^-Phe^6^, Phe^6^-Phe^7^, Trp^8^-Lys^9^ and Thr^10^-Phe^11^; Figure 2, Table 1), were replaced one by one by a Tz (XG1, XG2, XG3 and XG4, respectively) with an additional substitution made adjacent to a known enzymatic cleavage site (Phe^11^-Thr^12^ in XG5). All analogs were functionalized with DOTA at the *N*-terminus for the radiolabeling with In-111, a metallic radionuclide suitable for diagnostic nuclear imaging using single-photon emission computed tomography (SPECT).

The radiolabeled triazolo-peptidomimetics were fully evaluated in vitro with special emphasis given on SST_2_R internalization and SST_1-5_R affinity profile. Additionally, the metabolic stability of [^111^In]In-XG1 in peripheral mice blood was determined and [^111^In]In-AT2S was used for direct comparison. Pilot studies were conducted in mice bearing hSST_2_R-positive tumors to evaluate the tumor-targeting properties of [^111^In]In-XG1. To better understand the involvement of NEP on the metabolic stability of these analogs, we compared the metabolic stability of investigated peptides without or during NEP-inhibition. The latter was accomplished by pretreatment of mice with Entresto^®^, a registered antihypertensive drug releasing the potent and selective NEP-inhibitor sacubitrilat in vivo [18,29,30,31,32,33].

## 2. Materials and Methods

### 2.1. Materials and Instrumentation

Reagents were purchased from Sigma-Aldrich (Buchs, Switzerland), Merck (Darmstadt, Germany), Acros Organics (Geel, Belgium), Fluorochem (Hadfield, United Kingdom), Novabiochem (Darmstadt, Germany), TCI (Zwijndrecht, Belgium) and Fluka (Buchs, Switzerland) and were used without further purification. Preloaded resins H-L-Cys(Trt)-2-chlorotrityl (CTC) (0.71 mmol/g) and H-L-Thr(*^t^*Bu)-2-CTC (0.84 mmol/g) and amino acids were bought from Iris Biotech (Marktredwitz, Germany) and DOTA-tris(*^t^*Bu) ester was purchased from Chematech (Dijon, France). [^111^In]InCl_3_ used for the radiolabeling of the peptide conjugates was acquired from Curium (Petten, The Netherlands). The loading of the H-l-Cys(Trt)-2-CTC resins was determined on an Agilent 8453 UV-visible spectroscopy system (Vienna, Austria) at a wavelength of 301 nm (ε = 7800 L·mol^−1^·cm^−1^). The concentration of the peptide solutions was determined on a NanoDrop Micro-UV/Vis Spectrophotometer (One/one^c^ Microvolume UV-VIS Spectrophotometer, ThermoFisher scientific, Vienna, Austria) at 280 nm (ε = 5625 L·mol^−1^·cm^−1^) [34]. RP-HPLC-MS analyses of the peptides were conducted at 220 nm on an Agilent 1260 Infinity II system (Vienna, Austria) equipped with a Flexible pump, a 1260 VWD UV-Vis detector and the LC/MSD system using the Acquity UPLC^®^ Peptide BEH C18 column (300 Å, 1.7 μm, 2.1 mm × 100 mm). H_2_O (0.1% formic acid (FA)) and MeCN (0.1% FA) were used as mobile phases; for applied gradients, see Supplementary Materials. The chiral-HPLC (high-performance liquid chromatography) measurements of the α-amino alkynes were performed on a Thermo Scientific Dionex Ultimate 3000 UHPLC system (Vienna, Austria) equipped with a diode array detector and using a Dr Maisch, ReproSil Chiral-NR column (8 μm, 4.6 mm × 250 mm, Tübingen, Germany). *N-*Hexane and isopropanol were used as mobile phases using gradient described in the Supplementary Materials. Γ-HPLC analyses of the radio-peptides were performed on a VWR Hitachi Chromaster (Vienna, Austria) equipped with the 5410 UV detector (detection at 220 nm) and the Elysia Raytest Gabi Nova radioactivity detector and using the Jupiter Proteo column (90 Å, 4 μm, 4.6 mm × 250 mm). The preparative-RP-HPLC purification of the triazolo-peptidomimetics was accomplished on an Agilent 1260 Infinity system (Vienna, Austria) equipped with the Xselect^®^ Peptide CSH^TM^ C18 OBD^TM^ Prep column (130 Å, 5 μm, 19 mm × 150 mm) using H_2_O (0.1% TFA) and MeCN (0.1% TFA) as mobile phases. HR-MS Bruker maXis^TM^ UHR ESI time of flight spectrometer (Vienna, Austria) was used for mass determination. The experimental results were compared to the theoretical mass and isotopic distribution. NMR measurements were recorded on a Bruker FT-NMR Avance III^TM^ 500 MHz spectrometer (Vienna, Austria) at 500.10 (^1^H) and 125.75 (^13^C) MHz at 298 K in CDCl_3_. The residual signal of CDCl_3_ was used as internal reference for the chemical shifts (ppm). The spin multiplicities were abbreviated as follows: s = singlet, d = doublet, t = triplet, q = quartet, m = multiplet, bs = broad signal. The values of the coupling constants (*J*) are given in hertz (Hz).

The octapeptide TATE used In uptake and saturation binding assays was synthesized using the H-l-Thr(*^t^*Bu)-2-CTC resin following conventional Fmoc/*^t^*Bu SPPS. The HPLC-MS characterization of TATE can be found in the Supplementary Materials (Supplementary Figures S31 and S32).

The radiolabeling with [^111^In]InCl_3_ was conducted in low protein binding Eppendorf tubes. The neoBlock heater with inserts for Eppendorf tubes was used to accomplish the reaction. Alternatively, the radiolabeling reactions were conducted using a water bath.

Membrane filtration for the competition binding assays was performed on a Brandel^®^ Cell Harvester (Adi Hassel Ingenieur Büro, Munich, Germany) using Whatman GF/B glass fiber filters soaked 2 h in advance in an aqueous solution of polyethylenimine (PEI, 1%).

Samples were measured for 1 min with a 50–500 keV energy window with the γ-counter 2480 Wizard2 (PerkinElmer, Waltham, MA, USA) or the Canberra Packard Cobra^TM^ Quantum U5003/1, Auto-Gamma^®^ counting system. Measurements were also conducted with an automated well-type γ-counter (an automated multisample well-type instrument with a NaI(Tl) 3” crystal, Canberra Packard Cobra^TM^ Quantum U5003/1, Auto-Gamma^®^ counting system; Little Rock, AR/USA).

Cell culture reagents were purchased from Thermo Fisher Scientific (Vienna, Austria) or Sigma-Aldrich (Vienna, Austria). The AR42J cell line was purchased from the American Type Culture Collection (ATCC, Manassas, VA, USA), while the CHO-K1 cell line transfected with the hSSt_1_R (CHO-hSSt_1_R) was obtained from the European Collection of Authenticated Cell Culture (ECACC, Braunschweig, Germany). HEK293 cells transfected to stably express each of hSST_2,3,5_R were kindly provided by Prof. S. Schultz (Jena, Germany).

### 2.2. Synthesis

The synthesis of the α-amino alkyne building block required for the insertion of Tz into the backbone of peptides was synthesized as previously reported. For a detailed description, see the Supplementary Materials. Manual SPPS was performed as previously reported [26]. In brief, the Cys(Trt)-preloaded-2-CTC resin was swollen and the amino acids were mounted by the general procedure 1 followed by Fmoc deprotection specified in general procedure 2. The incorporation of the triazole heterocycle into the peptide backbone was accomplished by a diazotransfer reaction (general procedure 3) followed by the CuAAC reaction (general procedure 4) with the corresponding α-amino alkynes (for complete synthesis and characterization, see Supplementary Materials, pp. 3–16). The peptide was elongated until completion of the DOTA-containing sequence and the cleavage, cyclization and full deprotection were performed in accordance with general procedure 5. Finally, the peptide was purified by preparative-RP-HPLC as described in general procedure 5 and characterized by mass spectrometry.

#### 2.2.1. General Procedure 1. Manual Solid-Phase Peptide Synthesis

Cys(Trt)-preloaded-2-CTC resin (42 mg, 0.71 mmol/g) was swollen in DMF (3 × 3 mL) in a syringe fitted with a polypropylene frit and a Teflon tap (Biotage^©^; Uppsala, Sweden). Fmoc-protected amino acids (0.06 mmol, 2 equiv.), HATU (0.06 mmol, 2 equiv.) and DIPEA (0.15 mmol, 5 equiv.) were dissolved in 3 mL of DMF and added to the suspension and the syringe was shaken for 2 h at room temperature (RT). The solvent was filtered off and the resin was washed using DCM (3 × 3 mL) and DMF (3 × 3 mL). The coupling of the DOTA chelator was conducted twice using DOTA-tris(*^t^*Bu)ester (0.09 mmol, 3 equiv.), HATU (0.135 mmol, 4.5 equiv.), DIPEA (0.24 mmol, 8 equiv.) in 3 mL DMF. In order to test the completion of coupling reactions, a Kaiser test was conducted after every coupling reaction [35]. In case of incomplete conversion, the coupling reaction was repeated.

#### 2.2.2. General Procedure 2. Fmoc Deprotection on Resin

Three cycles of Fmoc-deprotection were conducted by adding 3 mL of 20% piperidine in DMF (3 mL) to the resin. After filtration, the resin was washed with DCM (3 × 3 mL) and DMF (3 × 3 mL). The yield was calculated based on the UV titration (λ= 301 nm) of the fluorenylmethylpiperidine adduct in the filtrate (ε = 7800 mol^−1^dm^3^cm^−1^) [36].

#### 2.2.3. General Procedure 3. On Resin Azido Functionalization of Peptide *N-*Terminus

A solution of ISA·HCl (0.15 mmol, 5 equiv.), and DIPEA (0.18 mmol, 6 equiv.) in DMF (3 mL) was added to the resin. The mixture was shaken for 2 h at RT. The solvent was removed by filtration and the resin washed with DCM (3 × 3 mL) and DMF (3 × 3 mL). The presence of the azido functional group was determined by the Punna–Finn colorimetric test [37]. 

#### 2.2.4. General Procedure 4. On Resin Copper(I) Catalyzed Alkyne Azide Cycloaddition (CuAAC)

To the resin with the sequence containing the azide at the *N-*terminus, a solution of the corresponding Fmoc-protected amino alkyne (0.06 mmol, 2 equiv.), DIPEA (0.03 mmol, 1 equiv.), [Cu(CH_3_CN)_4_]PF_6_ (0.015 mmol, 0.5 equiv.) and TBTA (0.015 mmol, 0.5 equiv.) in 3 mL of DMF (3 mL) was added. The suspension was shaken for 12–15 h at RT. The solvent was filtrated off and the resin thoroughly washed with 0.5% sodium diethyldithiocarbamate solution in DMF (3–5 × 3 mL) followed by DMC (3 × 3 mL) and DMF (3 × 3 mL). The presence of azido groups was checked by the Punna–Finn test and the completion of the CuAAC reaction determined by the Kaiser test. 

#### 2.2.5. General Procedure 5. Peptide Cleavage, Cyclization, Complete Deprotection and Purification

To cleave the peptide from the resin and selectively remove the side-chain protecting groups of the cysteine (Cys) residues, 3 mL of a cleavage cocktail (DCM/TFE/AcOH 7:2:1) were added to the syringe containing the resin with the full sequence. After shaking for 2 h at RT, the solution was added over a 0.8 M iodine (0.3 mmol, 10 equiv.) solution in a DCM/TFE/AcOH (7:2:1) mixture. The cyclization proceeded for 20–30 min at RT and the formation of the disulfide bridge was checked by HPLC-MS. The reaction was quenched with a 1 M ascorbic acid aqueous solution (2 mL) and extracted with DCM (3 × 3 mL). The organic phases were combined and washed with an aqueous 5% NaCl/citric acid (1:1) solution (3 × 3 mL). The organic phase containing the product was concentrated under reduced pressure and 4 mL of a cocktail containing TFA/TIPS/H_2_O (9.5:0.25:0.25) was added for the removal of the remaining side-chain protecting groups. The reaction was let to stir for 4 h at RT and the resulting solution was washed with heptane (3 × 2 mL) and concentrated with a stream of argon. The peptide was precipitated with MTBE (10 mL), vortexed, sonicated and centrifuged (4000 rpm, 5 min). The crude peptides (typically 10 mg) were dissolved in 1 mL of a H_2_O/MeCN (80:20) mixture containing 0.1% TFA and purified by preparative-HPLC eluted at a flow rate of 17 mL/min with the linear gradient from 80% A/20% B to 40% A/60% B in 30 min (A: 0.1% aqueous TFA and B: MeCN). The purification afforded the peptides in moderate yields (10–20%) and excellent purities (>95%).

#### 2.2.6. De Novo Synthesis of AT2S

The all-amide-bond reference compound AT2S was synthesized at a scale of 0.030 mmol following the general procedures 1, 2, 5 and 6 using the commercially available amino acids Fmoc-Ser(*^t^*Bu)-OH, Fmoc-Thr(*^t^*Bu)-OH, Fmoc-Phe-OH, Fmoc-Lys(Boc)-OH, Fmoc-d-Trp(Boc)-OH, Fmoc-Asn(Trt)-OH, Fmoc-Gly-OH, Fmoc-Ala-OH and DOTA-tris(*^t^*Bu)-ester. The peptide was obtained in high purity (>95%, Figures S17 and S18) as a white fluffy solid.

[M+2H]^2+^, [M+3H]^3+^ and [M+4H]^4+^ were calculated for C_92_H_130_N_22_O_26_S_2_: 1012.9571, 675.6420, 506.9822, respectively. The results were 1012.9581, 675.6414 and 506.9830.

#### 2.2.7. Synthesis of XG1

Peptide conjugate XG1 was synthesized at a scale of 0.030 mmol following general procedures 1–6 using the commercial amino acids Fmoc-Ser(*^t^*Bu)-OH, Fmoc-Thr(*^t^*Bu)-OH, Fmoc-Phe-OH, Fmoc-Lys(Boc)-OH, Fmoc-d-Trp(Boc)-OH, Fmoc-Cys(Trt)-OH, Fmoc-Gly-OH and Fmoc-Ala-OH and DOTA-tris(*^t^*Bu)-ester. The synthesized α-amino alkyne Fmoc-Asn(Trt)-CCH was used for the triazole incorporation *via* the CuAAC reaction. The peptide was obtained in high purity (>95%, Figures S19 and S20) as a white fluffy solid.

[M+2H]^2+^, [M+3H]^3+^ and [M+4H]^4+^ were calculated for C_93_H_130_N_24_O_25_S_2_: 1024.9627, 683.6440 and 512.9850, respectively. The results were 1024.9629, 683.6449 and 512.9857.

#### 2.2.8. Synthesis of XG2

Peptide conjugate XG2 was synthesized at a scale of 0.030 mmol following the general procedures (1–6) using the Fmoc-Ser(*^t^*Bu)-OH, Fmoc-Thr(*^t^*Bu)-OH, Fmoc-Phe-OH, Fmoc-Lys(Boc)-OH, Fmoc-d-Trp(Boc)-OH, Fmoc-Asn(Trt)-OH, Fmoc-Cys(Trt)-OH, Fmoc-Gly-OH and Fmoc-Ala-OH commercially available amino acids and the DOTA-tris(*^t^*Bu)-ester. The synthesized Fmoc-Phe-CCH amino alkyne was used for the triazole incorporation *via* the CuAAC reaction. The peptide was obtained in high purity (>95%, Figures S21 and S22) as a white fluffy solid.

[M+2H]^2+^, [M+3H]^3+^ and [M+4H]^4+^ were calculated for C_93_H_130_N_24_O_25_S_2_: 1024.9627, 683.6440 and 512.9850, respectively. The results were 1024.9634, 683.6453 and 512.9859.

#### 2.2.9. Synthesis of XG3

Peptide conjugate XG3 was synthesized at a scale of 0.030 mmol following the general procedures (1–6) using the Fmoc-Ser(*^t^*Bu)-OH, Fmoc-Thr(*^t^*Bu)-OH, Fmoc-Phe-OH, Fmoc-Lys(Boc)-OH, Fmoc-Asn(Trt)-OH, Fmoc-Cys(Trt)-OH, Fmoc-Gly-OH and Fmoc-Ala-OH commercially available amino acids and the DOTA-tris(*^t^*Bu)-ester. The synthesized Fmoc-d-Trp(Boc)-CCH amino alkyne was used for the triazole incorporation *via* the CuAAC reaction. The peptide was obtained in high purity (>95%, Figures S23 and S24) as a white fluffy solid.

[M+2H]^2+^, [M+3H]^3+^ and [M+4H]^4+^ were calculated for C_93_H_130_N_24_O_25_S_2_: 1024.9627, 683.6440 and 512.9850, respectively. The results were 1024.9619, 683.6443 and 512.9852.

#### 2.2.10. Synthesis of XG4

Peptide conjugate XG4 was synthesized at a scale of 0.030 mmol following the general procedures (1–6) using the Fmoc-Ser(*^t^*Bu)-OH, Fmoc-Thr(*^t^*Bu)-OH, Fmoc-Phe-OH, Fmoc-Lys(Boc)-OH, Fmoc-d-Trp(Boc)-OH, Fmoc-Asn(Trt)-OH, Fmoc-Cys(Trt)-OH, Fmoc-Gly-OH and Fmoc-Ala-OH commercially available amino acids and the DOTA-tris(*^t^*Bu)-ester. The synthesized Fmoc-Thr(*^t^*Bu)-CCH amino alkyne was used for the triazole incorporation *via* the CuAAC reaction. The peptide was obtained in high purity (>95%, Figures S25 and S26) as a white fluffy solid.

[M+H]^2+^, [M+3H]^3+^ and [M+4H]^4+^ were calculated for C_93_H_130_N_24_O_25_S_2_: 1024.9627 and 683.6440, 512.9850, respectively. The results were 1024.9620, 683.6442 and 512.9850.

#### 2.2.11. Synthesis of XG5

Peptide conjugate XG5 was synthesized at a scale of 0.030 mmol following the general procedures (1–6) using the Fmoc-Ser(*^t^*Bu)-OH, Fmoc-Thr(*^t^*Bu)-OH, Fmoc-Phe-OH, Fmoc-Lys(Boc)-OH, Fmoc-d-Trp(Boc)-OH, Fmoc-Asn(Trt)-OH, Fmoc-Cys(Trt)-OH, Fmoc-Gly-OH and Fmoc-Ala-OH commercially available amino acids and the DOTA-tris(*^t^*Bu)-ester. The synthesized Fmoc-Phe-CCH amino alkyne was used for the triazole incorporation *via* the CuAAC reaction. The peptide was obtained in high purity (>95%, Figures S27 and S28) as a white fluffy solid.

[M+2H]^2+^, [M+3H]^3+^ and [M+4H]^4+^ were calculated for C_93_H_130_N_24_O_25_S_2_: 1024.9627, 683.6440 and 512.9850, respectively. The results were 1024.9635, 683.6451 and 512.9857.

#### 2.2.12. Preparation of ^nat^In-XG1

For the determination of the IC_50_ values, XG1 was complexed with nonradioactive indium(III). The peptide-conjugate (0.5 mg, 0.25 μmol) was dissolved in 250 μL of H_2_O and a ^nat^InCl_3_ (50 μL, 2.5 μmol, 0.05 M) solution in HCl 0.05 M mixed with 60 μL of a 0.3 M aq. NaOAC buffer solution was added. The final pH (4.5–5) of the solution was determined using pH stripes. The reaction was heated for 20 min at 95 °C in a heating block and the completion of the reaction was monitored by HPLC-MS. The metal-tagged conjugate was purified with a SEP-PAK^®^ C-18 cartridge (conditioned with 1 mL of EtOH followed by 3 mL of H_2_O). Following the conditioning, the reaction mixture was loaded and the unbound ^nat^InCl_3_ was washed out with 3 mL of H_2_O. ^nat^In-XG1 was eluted with a H_2_O/MeCN (50/50) mixture (2 mL). The samples were lyophilized yielding a white solid (80%) of high purity (>95%, Figures S29 and S30).

[M+2H]^2+^, [M+3H]^3+^ and [M+4H]^4+^ were calculated for C_93_H_130_N_24_O_25_S_2_In: 1080.9035 and 720.9377, respectively. The results were 1080.9044, 720.9390 and 540.9559.

### 2.3. Radiolabeling with [^111^In]InCl_3_

Aliquots of the purified peptides were prepared at a concentration of 1 μM by dissolving 1 mg of peptide in Millipore Water/MeCN (9/1). The concentration of the peptides was determined at 280 nm (ε = 5625 L·mol^−1^·cm^−1^) with the Nanodrop Micro-UV/Vis system. The radiolabeling solution was prepared by mixing 50 μL of [^111^In]InCl_3_ (20–30 MBq) in 0.050 M HCl, 10 μL of aq. NaOAc buffer (0.3 M) and 5 μL of peptide solution (1 μM) with final pH values of 4.2–4.5 (determined with pH stripes, Macherey Nagel^©^, Düren, Germany). The radiolabeling of the DOTA-peptide conjugates was carried out by incubation at 95 °C for 10–15 min. Samples for quality control were prepared by mixing 1 μL of the labeling solution with 1 μL of a DTPA solution and 40 μL of Millipore Water (+0.1% TFA). The quality control was achieved by γ-HPLC at a flow rate of 3 mL/min on a Jupiter Proteo column (90 Å, 4 μm, 4.6 mm × 250 mm) with the linear gradient from 90% A/10% B to 50% A/50% B in 14 min (A: 0.1% aqueous TFA and B: MeCN). The radiopeptides were obtained in >95% radiochemical yields and radiochemical purity of >95% and used for in vitro and in vivo experiments without further purification (see Supplementary Materials for the γ-HPLC chromatograms, Figures S33–S38).

### 2.4. In Vitro Studies

#### 2.4.1. Cell Culture

All the cells were cultured in T75 or T175 Cellstar^®^ cell culture flasks with a filter cap in humidified air at 37 °C and 5% CO_2_. The SST_2_R-expressing rat pancreatic cells (AR42J) were cultured in Dubelco’s Modified Eagle’s Medium (DMEM), containing Glutamax-1. The medium was supplemented with 10% (*v*/*v*) fetal bovine serum (FBS), animal-free recombinant human EGF (2 ng/mL), human insulin (4 μg/mL) and 1% (*v*/*v*) penicillin-streptomycin (10,000 U/mL). The HEK293 cells, transfected to stably express each of the hSST_2,3,5_R with the T7 epitope, were cultured in DMEM supplemented with Glutamax-1 and 10% (*v*/*v*) FBS, 1% (*v*/*v*), penicillin-streptomycin (100 U/mL), and 400–500 µg/mL G418-sulfate. The CHO-K1 cells stably expressing the hSST_1_R receptor were cultured in Ham’s F-12 medium supplemented as described for transfected HEK293 cells. The membranes were prepared as previously reported and validated with the universal pansomatostatin ligand LTT-SST-28.

#### 2.4.2. Cell Binding and Internalization Assays and Blocking Experiments with AR42J Cells

AR42J cells were seeded in six-well plates (1 × 10^6^ cells/well) 2 days prior to the internalization assay resulting in 70–80% confluency. On the day of the experiment, the cells were counted with the LUNA^TM^ Automated cell counter (approximately 2 × 10^6^ cells/well). One hour before the experiment, the medium was removed, the cells were washed with PBS (1 × 1 mL) and fresh internalization medium (DMEM with Glutamax-1 and supplemented with 1% FBS (*v*/*v*), 1.3 mL/well) was added. The radiolabeled peptide was added to each well (0.250 pmol, 5 KBq, in 100 μL of PBS). For the determination of the nonspecific binding, blocking experiments (Figures S39 and S40) were conducted by adding a 1000-fold excess of TATE (250 pmol in 100 μL PBS) together with the radioligand. Next, the cells were incubated in culture conditions (37 °C, 5% CO_2_) for 10, 30, 60 and 120 min; the medium was removed followed by two washes with PBS (1 mL). The collected supernatants correspond to the nonbound fraction of the radiotracers. For the determination of the membrane-bound fraction, the six-well plates were placed on ice and washed twice for 5 min with 1 mL of ice-cold acidic glycine solution (100 mM NaCl, 50 mM glycine, pH 2.8). The cells were then lysed upon incubation with a 1 M NaOH aqueous solution (1 mL) for 10 min and the wells were washed with the NaOH solution (2 × 1 mL) representing the internalized fraction. Standards were prepared in triplicates by adding 0.250 pmol (5 KBq) of the radiopeptide in PBS (100 μL) to 1.4 mL of PBS. All collected fractions were measured in a γ-counter. Experiments were conducted in triplicates (n = 3) and data was analyzed using GraphPad Prism 8.0^TM^

#### 2.4.3. Receptor Saturation Binding Assay with SST_2_R-Expressing in AR42J Cells

AR42J cells were seeded in six-well plates (1 × 10^6^ cells/well) 2 days before the assay. One hour prior to the experiment, the medium was removed, the cells were washed with PBS (1 mL) and fresh assay medium (DMEM with Glutamax-1 and supplemented with 1% FBS (*v*/*v*)) was added (1.3 mL/well). The study of the [^111^In]In-AT2S reference was conducted by adding the radioligand in 100 μL of PBS at different concentrations (1, 10, 25, 50, 75, 100 nM). For [^111^In]In-XG1 and [^111^In]In-XG2 a different concentration range was chosen (10, 30, 60, 90, 120, 150 nM). The determination of the unspecific binding was conducted by incubating each test radioligand in the presence of excess TATE, which was added in 100 μL of PBS to a final concentration of 5 µM. The six-well plates were incubated under standard culturing conditions for 2 h; the medium was removed, the cells were washed with PBS (2 × 1 mL) and lysed as described above. The cell lysates were collected and measured in the γ-counter. The dissociation constant (K_D_) was calculated by nonlinear regression using the GraphPad Prism 8.0^TM^ program (n = 2–3 in triplicates).

#### 2.4.4. Competition Binding Assays in hSST_1,2,3,5_R Cell Membrane Homogenates

Competition binding assays were conducted for XG1 and ^nat^In-XG1 on cell membranes from each of HEK293-SST_2,3,5_R and CHO-K1-SST_1_R cells against the pansomatostatin radioligand [^125^I][I-Tyr^25^]LTT-SST-28 [38]. The experiments were conducted by adding 30 μL of XG1 or ^nat^In-XG1 at increasing concentrations (10^−13^–10^−5^ M), 30 μL of [^125^I][I-Tyr^25^]LTT-SST-28 (50 pM corresponding to approx. 40,000 cpm) and the respective membrane homogenate in binding buffer (200 µL, 50 mM HEPES at pH 7.4, 1% BSA, 5.5 mM MgCl_2_, 35 μM bacitracin) to each assay tube. After an incubation period of 1 h at 22 °C, the reaction was stopped by dilution with ice-cold washing buffer (10 mM HEPES at pH 7.4, 150 mM NaCl, 3 mL). The samples were filtered through 1%PEI presoaked glass fiber filters using a Brandel^®^ cell harvester. After thorough washing with ice-cold washing buffer (2 × 3 mL), the filters were collected and measured in the γ-counter. The IC_50_ (50% inhibitory concentration) values were calculated with GraphPad Prism 8.0^TM^ by normalized nonlinear regression (n = 3–4 in triplicate, for membrane validation and representative competition experiments, see Figures S41–S51).

#### 2.4.5. In Vitro Blood Plasma Stability Studies

Plasma stability studies were conducted in vitro by incubating the radioligands (in 100 µL PBS) in 1400 µL of human blood plasma (H4522–20ML, Sigma-Aldrich, Vienna, Austria) to a final concentration of 250 nM (approximately 5 MBq). After an incubation period of 6 h at 37 °C, aliquots of 100 µL were mixed with 100 µL of MeCN for protein precipitation. The mixtures were vortexed and centrifuged for 10 min (15,000× *g*) at 5 °C and the supernatants were collected. The supernatants (50 µL) were mixed with 0.1% aqueous TFA (50 µL) for posterior γ-HPLC analyses on a Chromolith^®^ RP-18 performance column (2 µm, 4.6 mm × 100 mm; Merck Millipore, Vienna, Austria) eluted at a flow rate of 3 mL/min with the linear gradient from 90% A/10% B to 50% A/50% B in 14 min (A: 0.1% aqueous TFA and B: MeCN) (n = 2).

### 2.5. In Vivo Studies

All mice experiments were conducted in licensed facilities (EL 25 BIO exp021) and complied with European and national regulations. The study protocols were approved by the Department of Agriculture and Veterinary Service of the Prefecture of Athens (#1609, 24 April 2019 for the stability studies and #1610, 24 April 2019 for the biodistribution studies).

#### 2.5.1. Metabolic Stability Studies

Healthy male Swiss albino mice (>8 weeks of age, body weight: 30 ± 5 g) provided by the NCSR “Demokritos” Animal House (Athens, Greece) were used in the in vivo stability studies. Animals (3 per compound administered with a 100 μL bolus (saline/EtOH 9/1 *v*/*v*) containing the test radioligand (11–22 MBq, 3 nmol) *via* the tail vein either without pretreatment (controls) or 20–30 min following *per os* administration of Entresto^®^ (12 mg/200 µL per animal, Entresto^®^ groups). Individual Entresto^®^ doses were prepared from Entresto^®^ pills purchased from a local pharmacy (200 mg corresponding to 24 mg/26 mg sacubitril/valsartan per pill), containing the prodrug sacubitril (Novartis, Basel, Switzerland) [39]. The pills were ground to a fine powder in a mortar, divided and suspended in tap water to the desired individual doses, as previously reported [29]. Mice were sacrificed 5 min pi and blood was withdrawn from the heart in a precooled syringe. The blood was immediately transferred in a low protein binding Eppendorf tube on ice containing 40 μL of an aqueous solution of ethylenediaminetetraacetic acid (EDTA, 50 mM Na_2_EDTA). Samples were centrifuged for 10 min at 4°C (2000× *g*), the supernatant was separated and an equal volume of MeCN was added for protein precipitation. After a centrifugation period of 10 min at 4 °C (15,000× *g*), the supernatant was collected and concentrated under a gentle stream of N_2_ at 40 °C to an approximate volume of 50–100 μL. Physiological saline was added (400 μL) and the solution was filtered through a Millex GV filter. The samples were analyzed by γ-HPLC to detect radiometabolites formed in vivo. For analyses on a Symmetry Shield RP18 cartridge column (5 μm, 3.9 mm × 20 mm; Waters, Eschborn, Germany) eluted at a flow rate of 1 mL/min with the linear gradient from 100% A/0% B to 60% A/40% B in 40 min (A: 0.1% aqueous TFA and B: MeCN). The r_t_ (retention time) of intact radioligand in this system was verified by co-injecting the serum sample with an aliquot of the labeling solution (HPLC chromatograms can be found in Supplementary Materials, Figure S54).

#### 2.5.2. SPECT/CT Imaging and Ex Vivo Determination of Tumor/Kidney Uptake

Three male severe combined immunodeficiency (SCID) mice (23.1 ± 1.6 g body weight, 6 weeks of age on arrival day; NCSR “Demokritos” Animal House, Athens, Greece) were used for SPECT/CT imaging. Animals were subcutaneously inoculated with a sterile suspension of freshly harvested HEK293-hSST_2_R (1.2 × 10^7^ cells) in the right flank and with wtHEK293 cells (0.6 × 10^7^ cells) in the left flank (negative control). After 4 weeks, the mice developed well-palpable tumors at the implantation sites. During this period, mice were housed in suitable facilities under aseptic conditions with 12 h day/night cycles and were provided with sterilized chow food and drinking water ad libitum. At the date of the experiment, one untreated animal received a bolus of [^111^In]In-XG1 (100 μL, 3 nmol in vehicle: normal saline/EtOH 9/1 *v*/*v*, control, 11–22 MBq) through the tail vein, while one mouse received per os Entresto^®^ (100 μL, 12 mg; Entresto^®^) 30 min in advance. Mice were euthanized at 4 h pi and SPECT/CT images were obtained on a x-CUBE & γ-CUBE (Molecubes, BIOMTECH, Athens, Greece) instrument. For SPECT, 40 min acquisition at 4 pi, MLEM (maximum-likelihood expectation maximization) reconstruction with 250 µm voxel size and 100 iterations protocol was adopted, while for CT acquisition, a high-resolution protocol (50 kVp), ISRA reconstruction with 100 um voxel size was applied, as previously described. After completion of the imaging experiment, mice were dissected and the implanted tumors as well as the kidneys were excised, weighed and measured for radioactivity in the γ-counter together with proper standards of the injected dose. Results were calculated as percentage of injected activity per gram tissue (%IA/g, see the Figure S56 detailed results).

## 3. Results and Discussion

### 3.1. Synthesis and (Radio)Metal Labeling of Triazolo-Peptidomimetics

The *trans*-amide bonds subjected to the amide-to-triazole substitution were selected based on the enzymatic cleavage sites reported for SST-14. Thus, the zinc-dependent endopeptidase NEP has been identified as the key degrading protease of native SST-14, cleaving several *trans*-amide bonds within the 12mer cycle of the 14peptide backbone (Figure 2) [19,40,41]. These bonds were substituted by a Tz and in addition the Phe^11^-Thr^12^ bond adjacent to a NEP cleavage site was also replaced (XG5). The inclusion of a Tz in a position adjacent to a cleavage site has been reported to effectively enhance the metabolic stability of NEP-substrates [29].

**Scheme 1:**
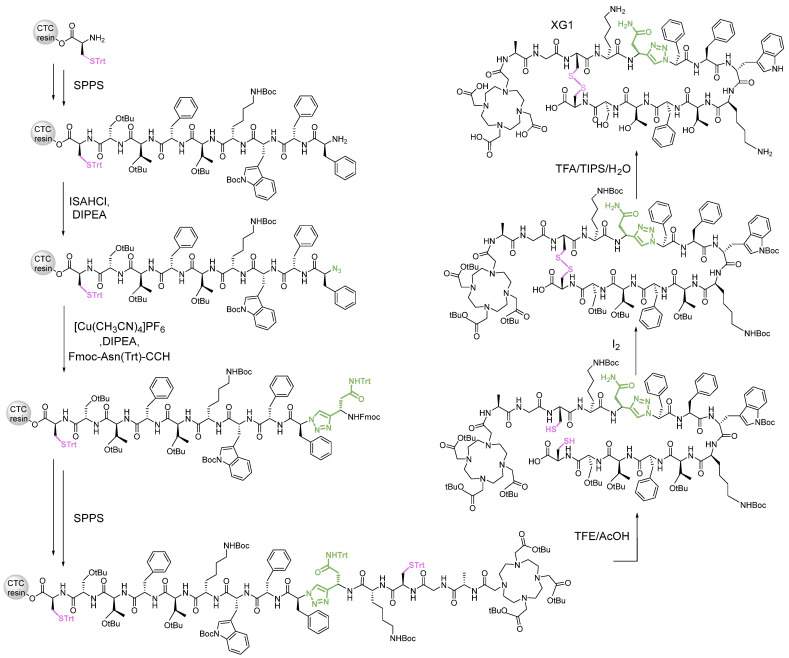
Synthesis of the 1,4-Tz-containing peptidomimetic XG1 with the side-chains of the cysteine residues used in the cyclization in magenta and the Tz in olive-green. CTC refers to the 2-chlorotrityl resin.

The synthesis of five cyclic triazolo-peptidomimetic AT2S analogs was accomplished by adapting protocols previously described (Scheme 1, Table 1) [26]. In brief, following the orthogonal Fmoc/^t^Bu SPPS, the 1,4-disubstituted 1,2,3-Tz was inserted in the peptide backbone in two steps. First, the Fmoc protecting group was removed from the *N-*terminal amine and the amide-to-azide conversion was accomplished using the imidazole-1-sulfonyl azide hydrochloride (ISA·HCl) as diazotransfer reagent [42]. The subsequent copper(I) catalyzed azide-alkyne cycloaddition (CuAAC) click reaction was conducted using a suitable α-amino alkyne derivative of the next amino acid in the sequence employing tetrakis(acetonitrile)copper(I) hexafluorophosphate ([Cu(CH_3_CN)_4_]PF_6_) as catalyst [43]. The amino acid sequence was then further elongated and completed by appending the DOTA chelator to the *N-*terminal amine of the peptide. The peptide was cleaved from the resin and the trityl protecting groups of the two cysteine residues were selectively removed with a cocktail containing acetic acid and trifluoroethanol (TFE). The disulfide-bond driven cyclization was accomplished using an excess of iodine and the remaining protecting groups were subsequently removed by treatment with a cocktail containing trifluoroacetic acid (TFA) and scavengers. Purification by HPLC yielded the triazolo-peptidomimetics in excellent purities (>95%, see supporting information (Supplementary Materials) for complete synthetic scheme and HPLC as well as for mass spectrometry (MS) data, Figures S17–S30) demonstrating for the first time that the amide-to-triazole switch methodology is applicable to the synthesis of cyclic triazolo-peptidomimetics (Figure 1, Table 1). The obtained five DOTA-conjugates were radiolabeled with [^111^In]InCl_3_ in radiochemical purities and yields (RCP, RCY) of >95% (Figures S33–S38) at molar activities of 3.7–7.3 MBq/nmol (decay corrected) and were used in vitro and in vivo without further purification. The synthesis of non-radioactive ^nat^In-XG1 was accomplished in a NaOAc buffer by mixing ^nat^InCl_3_ with an aqueous solution of XG1 (final pH = 4.5–5). Following purification *via* a Sep-PAK^®^ C-18 cartridge, ^nat^In-XG1 was isolated with high purity (>95%) and yield (80%).

### 3.2. In Vitro Evaluation

**Figure 3:**
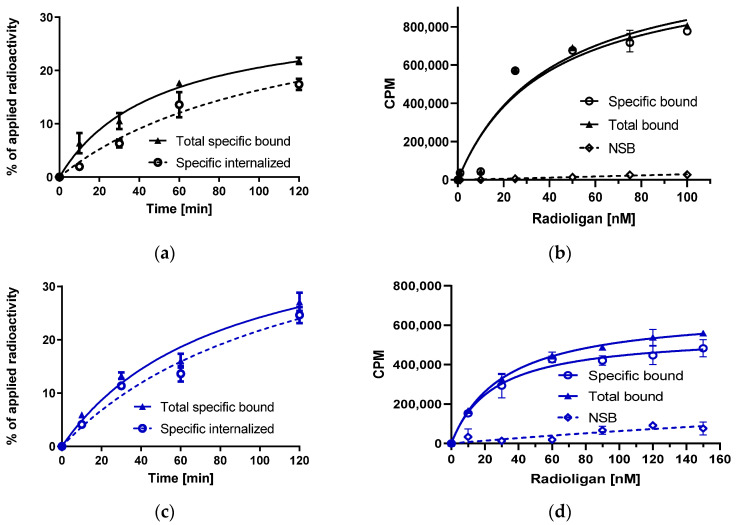
In vitro characterization of (**a**,**b**) [^111^In]In-AT2S and (**c**,**d**) [^111^In]In-XG1 in SST_2_R-expressing AR42J cells where cell binding and internalization kinetics were studied (**a**,**c**, n = 3 in triplicates) and (**b**,**d**) the K_D_ were determined in saturation binding assays (n = 2–3 in triplicates) using GraphPad Prism 8.0. Data points show mean values ± standard deviation (SD). NSB stands for nonspecific binding.

The ^111^In-radioligands were tested in vitro for their cell binding and internalization properties first for the SST_2_R subtype, due to its predominant expression across a wide range of tumors [10]. Thus, only compounds with a retained high affinity toward the SST_2_R were considered for further investigations. For this purpose, the rat SST_2_R-expressing AR42J cell line was used. To verify SST_2_R-specificity of the radiolabeled peptides, a 1000-fold excess of the SST_2_R-prefering TATE was used for receptor blocking experiments (Figures S31 and S32). In accordance with reported data, a rapid and SST_2_R specific cell association and internalization was observed for [^111^In]In-AT2S (Figure 3a) with 22% of the applied activity (A.A) was found associated to the cells after 2 h, from which 80% (17% A.A) corresponds to the internalized fraction [15]. A similar trend was observed for [^111^In]In-XG1, which displayed an equally fast but superior cell binding and internalization capacity (24% of A.A was internalized, Figure 3). During receptor blockade in the presence of an excess TATE, minimal binding and internalization (1% A.A) of the radioligands were observed, thus confirming their SST_2_R-specificity. In case of [^111^In]In-XG2, the cell associated radioactivity was found to be considerably less (6% A.A). Given the low SST_2_R-specific cell associated activity observed for [^111^In]In-XG3, XG4 and XG5 (< 1% A.A), they were excluded from further studies. We hypothesize that the proximity of the introduced Tz-modifications to the pharmacophore of the peptide (Phe^7^-d-Trp^8^-Lys^9^-Thr^10^) resulted in structural changes, detrimental for interaction with the SST_2_R [44].

Given the retained ability of [^111^In]In-XG1 and [^111^In]In-XG2 to internalize *via* SST_2_R, we set out to determine their affinity toward this receptor. Thus, SST_2_R-saturation studies were conducted by incubation of [^111^In]In-XG1 and [^111^In]In-XG2 at increasing concentrations in AR42J cells (Figure 3) and the KD of each compound was determined (Figure 3). Interestingly, despite the superior internalization of [^111^In]In-XG1, the [^111^In]In-AT2S reference displayed higher affinity toward SST_2_R (KD = 12.04 ± 3.88 nM, Figure 3a) compared to the triazolo-peptidomimetic (KD = 25.11 ± 4.32 nM, Figure 3b). This partial loss of affinity for SST_2_R was more pronounced for [^111^In]In-XG2, the KD value of which was found to be much higher (>50 nM), compromising its prospects for clinical applicability. Consequently, only [^111^In]In-XG1 was selected for further in vitro experiments to determine the SST_1-3,5_R affinity profile.

**Figure 4:**
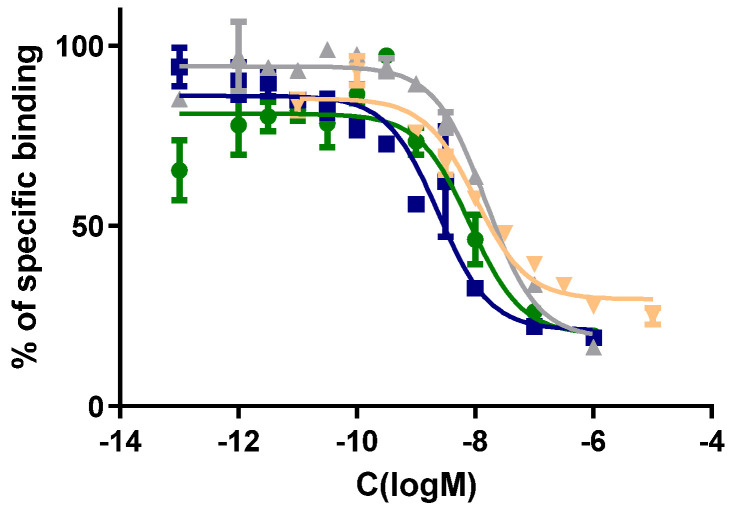
Displacement of [^125^I][I-Tyr^25^]LTT-SST-28 from hSST_1_R (yellow line), hSST_2_R (green line), hSST_3_R (blue line) and hSST_5_R (gray line)-expressing membranes at increasing concentrations of ^nat^In-XG1; by using GraphPad Prism 8.0., data points show mean values ± SD from four experiments performed in triplicate.

The pansomatostatin profile of [^111^In]In-XG1 was investigated by competition binding assays of XG1 and the ^nat^In-XG1 surrogate against the pansomatostatin [^125^I][I-Tyr^25^]LTT-SST-28 radioligand. The IC_50_ values were determined using membrane homogenates from cells transfected to stably express one of the hSST_1-3,5_R (Figure 4). hSST_4_R was not included in this study due to its minor relevance in oncology [3,12]. ^nat^In-XG1 exhibited a similar pansomatostatin profile when compared to AT2S, with slightly lower affinities for hSST_1_R and hSST_2_R (Table 2). The observed small reduction in the hSST_2_R-affinity is in line with the respective K_D_ values acquired during saturation binding assays in SST_2_R-positive AR42J cells. In addition, we investigated the affinity profile of the nonmetal-tagged XG1 in order to assess the influence of the metal on the binding affinity toward hSSTRs. XG1 and ^nat^In-XG1 have a similar affinity profile but they differ in the affinity toward hSST_1_R, where ^nat^In-XG1 displayed clearly superior affinity compared to nonmetal-tagged XG1 (Table 2). It is reported that GPCRs with a high degree of glycosylation can influence the binding affinities of chelator/metal-chelate-modified ligands due to distinct electrostatic interactions (this phenomenon has mainly been described for SST_2_R) [45]. It has been reported that sialic acid is present in somatostatin receptors, and this carbohydrate could be responsible for the aforementioned electrostatic repulsive interactions.

In vitro stability studies were conducted for [^111^In]In-AT2S and the [^111^In]In-XG1 in human plasma. Both were found to be highly stable over 6 h of incubation at 37 °C (>90% of intact radiopeptides). This result is in disagreement with the poor in vivo stability reported for [^111^In]In-AT2S (only 6% of intact peptide after 5 min in blood circulation in mice) [15]. It is reported that the zinc-dependent endopeptidase NEP, an ectoenzyme anchored on epithelial cells on vessel walls and major tissues/organs of the body but not in the solute, plays a pivotal role in the degradation of SST-14 and its analogs. Hence, its action is not shown in the in vitro plasma studies [19]. In order to have a reliable measure of the metabolism of the peptides, in vivo stability studies of [^111^In]In-XG1 were conducted in mice.

### 3.3. In Vivo Evaluation

**Figure 5:**
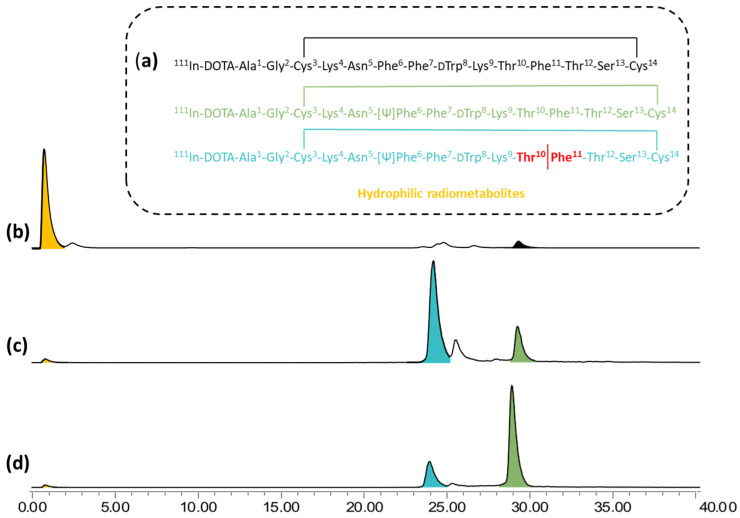
In vivo stability of (**a**) [^111^In]In-XG1. Color-coded structures of [^111^In]In-AT2S (black), [^111^In]In-XG1 (olive-green), the suggested major metabolite of [^111^In]In-XG1 (turquoise) and hydrophilic radiometabolites (yellow). The amino acids depicted in red represent an hydrolyzed amide bond. Analysis of blood sample 5 min pi of (**b**) [^111^In]In-AT2S (6% intact peptide), (**c**) [^111^In]In-XG1 (17% of intact peptide) and (**d**) [^111^In]In-XG1 from a mouse pretreated with Entresto^®^ (75% of intact peptide). Three mice were used per experiment.

The metabolic stability of [^111^In]In-XG1 in peripheral mice blood was compared to the rapidly metabolized [^111^In]In-AT2S in order to determine potential improvements in stability upon introduction of Asn^5^-*Ψ*[Tz]-Phe^6^ motif. Compared to the reference [^111^In]In-AT2S (6% intact peptide 5 min pi, Figure 5b), a significant improvement was achieved by [^111^In]In-XG1 (up to 17% of intact peptide, *p* < 0.0001, Figure 5c). It is interesting to note that the radiometabolite pattern notably differs between [^111^In]In-AT2S and [^111^In]In-XG1. Degradation of [^111^In]In-AT2S results in the formation of hydrophilic, likely low-molecular-weight metabolites r_t_ = approx. 0.5 min. Figure 5b) barely retained by the HPLC column. On the other hand, the major radiometabolite of [^111^In]In-XG1 (r_t_ = approx. 23 min) eluted close to the intact parent compound ([^111^In]In-XG1, r_t_ = approx. 28 min, Figure 5c) indicating the presence of a higher molecular weight metabolites. We hypothesize that the introduction of the Tz at the position Asn^5^-Phe^6^ of the peptide impedes with the complete cleavage of the pharmacophore of the peptide (Phe^7^-d-Trp^8^-Lys^9^-Thr^10^), thereby preventing further cleavage of amide bonds. In the case of [^111^In]In-AT2S, this process was not hampered resulting in the formation of hydrophilic metabolites (Figure 5b).

In order to assess the involvement of NEP in the metabolic fate of [^111^In]In-XG1, we compared the metabolic patterns of the radioligand injected alone (controls) or after pretreatment of mice with Entresto^®^ inducing in situ NEP-inhibition (Figure S53) [46]. As shown in Figure 5, [^111^In]In-XG1 appears to be considerably more stable in the Entresto^®^ group (75% of intact [^111^In]In-XG1 at 5 min pi). It is interesting to note that the same major radiometabolite is formed (r_t_ = approx. 23 min), albeit to a much lesser extent than in controls. These findings indicate that indeed NEP is the main peptidase responsible for the degradation of [^111^In]In-XG1 in blood. Based on these studies, we hypothesize that the Tz does not only impede with the cleavage of the Asn^5^-Phe^6^ bond, but also serves to prevent the hydrolysis of the amide between the Phe^6^-Phe^7^ bond. However, we infer that the amide bond between the Thr^10^-Phe^11^ residues remains vulnerable to the cleavage by NEP, which in turn could result in the formation of the radiometabolite observed at r_t_ 23 min (Figure 5a, turquoise structure). Since the introduction of a Tz in this position (Thr^10^-Phe^11^) was shown to be detrimental for SST_2_R-affinity, employment of alternative amide bond surrogates, in combination with the already incorporated Tz at Asn^5^-*Ψ*[Tz]Phe^6^, represents an interesting and promising alternative to enhance the stability in vivo while preserving the pansomatostatin profile of [^111^In]In-XG1. This work is currently ongoing and will be reported in due time.

**Figure 6:**
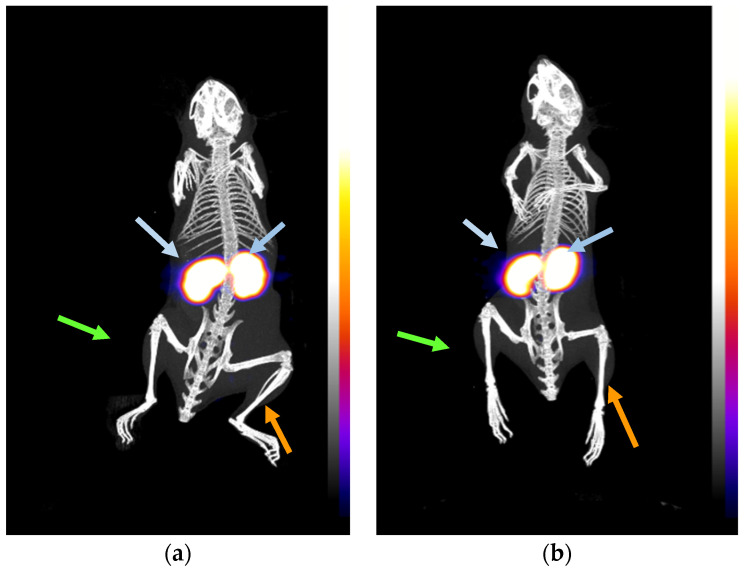
Static whole-body SPECT/CT images of SCID mice bearing HEK293-hSST_2_R tumors in their left flank (green arrows) and hSST_2_R-negative wtHEK293 control tumors in their right flank (orange arrow) at 4 h pi of [^111^In]In-XG1: (**a**) without pretreatment with Entresto^®^ or (**b**) 30 min following oral administration of Entresto^®^. The kidneys are indicated with blue arrows and the color bars represent the difference in accumulated activity (purple being the lowest and white the highest level of accumulation).

Next, a pilot SPECT/CT imaging study was conducted in two male SCID mice bearing HEK293-hSST_2_R (left flank) and control hSST_2_R-negative wtHEK293 (right flank) tumors. One animal served as untreated control, whereas the other was pretreated with Entresto^®^ as described above. At 4 h pi of [^111^In]In-XG1, animals were euthanized and static images were obtained (Figure 6). To quantify results, the tumors and the kidneys were resected, and the % IA/g values were measured on a γ-counter (Figure S56). In contrast to [^111^In]In-AT2S, [^111^In]In-XG1 exhibited a pronounced kidney uptake in untreated mice (>200% IA/g versus 23.20 ± 7.04% IA/g for [^111^In]In-AT2S [15]), which impeded with the visualization of the HEK293-hSST_2_R xenografts by SPECT/CT (Figure 6). The untreated mouse showed higher accumulation of [^111^In]In-XG1 in the HEK293-hSST_2_R tumor (1.61% IA/g) as compared to the SST_2_R-negative wtHEK293 control tumor (0.56% IA/g), demonstrating SST_2_R-specific uptake. However and despite the enhanced in vivo stability, the tumor uptake of [^111^In]In-XG1 was not superior to the reported data of [^111^In]In-AT2S (1.49 ± 0.2% IA/g) [15]. A direct comparison of the in vivo data should be drawn with caution because of different amounts of radiotracers applied (for this study: 11–22 MBq/3 nmol [^111^In]In-XG1 versus reported dose for [^111^In]In-AT2S of 34–74 KBq/10 pmol). The higher kidney uptake of [^111^In]In-XG1 could be attributed to its distinctive metabolism (Figure 5c). According to our hypothesis as outlined above, cleavage of the bond between Thr^10^-Phe^11^ amino acid residues would result in a radiometabolite that preserves two Lys residues (Lys^4^ and Lys^9^, Figure 5a). It is known that radiopeptides (or metabolites thereof) containing primary amines, such as in Lys residues, tend to accumulate in the kidneys. Interaction of the protonated amines under physiological conditions with the negatively charged glomerulus basement membrane is most probably key to this process [47]. A similar trend was observed for [^111^In-DOTA]LTT-SST-28, a pansomatostatin-like radiotracer with three Lys residues [12]. In the case of [^111^In]In-AT2S on the other hand, the absence of the Tz in the position Asn^5^-Phe^6^ would likely result in further degradation resulting in low molecular weight, hydrophilic metabolites lacking multiple positive charges and thus efficient clearance *via* the urine.

The mouse pretreated with Entresto^®^ showed an increased uptake of [^111^In]In-XG1 in the HEK293-hSST_2_R tumor (2.57% IA/g versus 1.61% IA/g for the untreated mice), confirming a positive outcome of this approach [18]. Yet, the use of Entresto^®^ did not alleviate the high renal uptake of [^111^In]In-XG1, in support of our hypothesis that the observed pronounced kidney uptake is a result of the intrinsic structural properties of the radiotracer. Different strategies to reduce the kidney uptake of radiolabeled peptides have been reported, some of which have found routine applications in the clinic. Examples include the infusion of solutions of basic amino acids (Lys, Arg) prior and/or during application of radiolabeled peptide or the use of plasma expanders [48,49,50]. Furthermore, the replacement of Lys by Arg was shown to effectively lead to reduced kidney uptake of radiolabeled peptides or metabolites thereof [50,51]. Finally, the use of cleavable linkers specific for enzymes present at the renal brush border membrane of kidneys have shown promising results [52,53]. Such approaches will be investigated in the future in order to improve the pharmacokinetic properties of our lead compound [^111^In]In-XG1.

## 4. Conclusions

We have applied the amide-to-triazole substitution strategy to the synthesis of five novel cyclic somatostatin-based triazolo-peptidomimetics. Only [^111^In]In-XG1 preserved the distinctive in vitro high binding affinity toward SST_1,2,3,5_R as in the case of reference compound [^111^In]In-AT2S, as well as demonstrating enhanced in vivo stability. When tested in mice bearing hSST_2_R-expressing tumors, the triazolo-peptidomimetic [^111^In]In-XG1 displayed receptor-specific tumor uptake. Yet, the introduction of the Tz in the peptide backbone had a marked effect on the uptake of radioactivity in the kidneys, which was found considerably higher for [^111^In]In-XG1 than for [^111^In]In-AT2S. We hypothesize that this is a result of the distinctive metabolism of [^111^In]In-XG1, which may lead to the formation of cationic radiometabolites that are trapped in the proximal tubule of the kidneys. The in vivo metabolic studies conducted with Entresto^®^, showed the efficacy of this antihypertensive drug at preserving the integrity of [^111^In]In-XG1 in blood through the inhibition of NEP. However, the pronounced kidney uptake was not influenced by the use of Entresto^®^ in tumor uptake studies. We therefore conclude that the use of [^111^In]In-XG1 could benefit from alternative strategies aiming at the reduction of renal uptake, such as the use of plasma expanders (e.g., Gelofusine^®^) or/and basic amino acid solutions. In addition, with the aim to further increase the metabolic stability of [^111^In]In-XG1, complementary structural modifications in combination with the triazole motif (Asn^5^-*Ψ*[Tz]-Phe^6^) are currently being explored.

## Figures and Tables

**Table 1:** HPLC-MS characterization of peptide and triazolo-peptidomimetic conjugates.

Compound ^1^	Structure ^2^	Purity [%] ^3^	m/z, [M+2H]^2+^ [Da] ^4,5^
AT2S	[DOTA,d-Trp^8^]SST-14	>95	1012.9581
XG1	[DOTA,Asn^5^-*Ψ*[Tz]-Phe^6^, d-Trp^8^]SST-14	>95	1024.9629
XG2	[DOTA,Phe^6^-*Ψ*[Tz]-Phe^7^, d-Trp^8^]SST-14	>95	1024.9634
XG3	[DOTA,D-Trp^8^-*Ψ*[Tz]-Lys^9^, d-Trp^8^]SST-14	>95	1024.9619
XG4	[DOTA,Thr^10^-*Ψ*[Tz]-Phe^11^, d-Trp^8^]SST-14	>95	1024.9620
XG5	[DOTA,Phe^11^-*Ψ*[Tz]-Thr^12^, d-Trp^8^]SS14	>95	1024.9635
^nat^In-XG1	[^nat^In-DOTA,Asn^5^-*Ψ*[Tz]-Phe^6^, d-Trp^8^]SST-14	>95	1080.9044

^1^ AT2S is the unmodified all-amide bond reference compound [15]; ^2^ *Ψ*[Tz] represents the *trans*-amide bond replaced by a 1,4-disubstituted 1,2,3-triazole; ^3^ purity was determined by reversed-phase HPLC; ^4^ molecular masses of peptides were measured by ESI-MS coupled to an HPLC system; ^5^ expected m/z of AT2S = 1012.9571, XG1–5 = 1024.9627 and ^nat^In-XG1 = 1080.9035.

**Table 2:** Affinity profiles of AT2S, XG1 and ^nat^In-XG1 for hSST_1-3,5_R; mean IC_50_ ± sd (in nanomolar), n = 4.

Peptide ^1^	hSST_1_R ^2^	hSST_2_R ^2^	hSST_3_R ^2^	hSST_5_R ^2^
AT2S [15]	14.0 ± 2.0	1.5 ± 0.3	2.4 ± 0.5	12.0 ± 2.0
XG1	81.7 ± 24.0	5.0 ± 3.5	3.0 ± 0.9	18.5 ± 6.0
^nat^In-XG1	23.1 ± 11.3	4.9 ± 3.5	1.8 ± 0.6	14.7 ± 1.6

^1^ AT2S refers to the unmodified reference and ^nat^In-XG1 to the triazolo-peptidomimetic XG1 tagged with natural indium; ^2^ membranes individually expressing each receptor subtype were used in competition binding assays.

## Data Availability

All data is contained within the article and the Supplementary Materials.

## Supplementary Materials

The following supporting information can be downloaded at: https://www.mdpi.com/article/10.3390/pharmaceutics16030392/s1, Figures S1–S16: NMR, HR-MS and chiral-HPLC characterization of α-amino alkynes; Figures S17–S32: HPLC-MS characterization of peptide conjugates; Figures S33–S38: γ-HPLC characterization of radio-conjugates; Figures S39 and S40: Binding and internalization studies; Figures S41–S52: Competition binding assays; Figure S53: Structures of the molecules contained in Entresto^®^; Figures S54 and S55: In vivo stability studies of [^111^In]In-XG and Figure S56: Tumor uptake studies of [^111^In]In-XG. Table S1. HPLC-MS characterization of peptide and triazolo-peptidomimetic conjugates (replica of Table 1 in the main manuscript). Scheme S1: R1 refers to amino acid specific side chain. R2 corresponds to the protected side chains of Asn, d-Trp, Thr and R3 corresponds to the side chain of Phe. (a) (i) amino acids, HATU, DIPEA (ii) piperidine; (b) ISA·HCl, DIPEA; Route 1 (c) N,O-dimethylhydroxylamine hydrochloride salt, BOP, DIPEA; (d) DIBAL-H; (e) Bestmann-Ohira reagent, K_2_CO_3_, MeOH; Route 2 (f) (S)-(-)-*tert*-butylsulfinamide, CuSO_4_; (g) *n*-BuLi, ethynyltrimethylsilane, AlMe_3_; (h) (i) TBAF (ii) HCl (iii) DIPEA, FmocOSu; (i) tetrakis(acetonitrile)Cu(I) hexafluorophosphate, DIPEA, TBTA.
