# Supplementary material for: Amide-to-Triazole Switch in Somatostatin-14-Based Radioligands: Impact on Receptor Affinity and In Vivo Stability
Source: Pharmaceutics. 2024 Mar 13;16(3):392. doi: 10.3390/pharmaceutics16030392 (PMC10976246; doi:10.3390/pharmaceutics16030392)
Supplement: Supplementary file 1 [file pharmaceutics-16-00392-s001.zip › pharmaceutics-2895638-supplementary.pdf]

Amide-to-triazole switch in somatostatin-14-based radioligands: Impact on receptor affinity and in vivo stability

Xabier Guarrochena [1–4] †, Panagiotis Kanellopoulos [5] †, Anna Stingeder [3,4], Lisa-Maria Rečnik [1,3,4], Irene V. J. Feiner [1,3,4], Marie Brandt [1,3,4], Wolfgang Kandioller [6], Theodosia Maina [5], Berthold A. Nock [5*], and Thomas L. Mindt [1,–4,7*]

1. Table of contents

1.	Table of contents	1
2.	Abbreviations	2
3.	Synthesis of building blocks	3
3.1	Synthesis of Weinreb amides	3
3.2	Synthesis of α-amino alkynes	3
3.3	Synthesis of n-sulfinyl imine	4
3.4	Synthesis of n-sulfinyl propargylamines	4
3.5	Desilylation of n-sulfinyl propargylamines	4
3.6	Sulfinamide cleavage and Fmoc protection	4
4.	Characterization of α-amino alkynes	5
5.	Characterization of triazolo-peptidomimetics	17
6.	γ-HPLC chromatograms	26
7.	Binding and internalization assays	29
8.	Competition binding assays	30
9.	Structure of Entresto® and in vivo activation of sacubitril	34
10.	Metabolic stability experiments	35
11.	Tumor/kidney uptake studies	36
12.	References	37

2. Abbreviations

BOP, (Benzotriazol-1-yloxy)tris(dimethylamino)phosphonium hexafluorophosphate; BuLi, butyllithium; CuAAC, Copper(I)-catalyzed Azide-Alkyne Cycloaddition; DCM, dichloromethane; DEPT, Distortionless Enhancement by Polarization Transfer; DIBAL-H, diisobutylaluminum hydride; DIPEA, N,N-diisopropylethylamine; DMF, N,N-dimethylformamide; DOTA, 1,4,7,10-tetraazacyclododecane1,4,7,10-tetraacetic acid; ESI-MS, electrospray ionization mass spectrometry; HATU, O-(7- azabenzotriazol-1-yl)-N,N,N',N'-tetramethyluronium hexafluorophosphate; HPLC, high-performance liquid chromatography; $hSST_2R$, human somatostatin receptor subtype 2; IPA, isopropanol; ISA·HCl, imidazole-1-sulfonyl azide hydrochloride salt; MeOH, methanol; NMR, nuclear magnetic resonance; RT, room temperature; r_t, retention time; TBAF, tetra-n-butylammonium fluoride; TBTA, tris[(1-benzyl-1H-1,2,3-triazol-4-yl)methyl]amine; THF, tetrahydrofuran; TLC, thin layer chromatography; TFA, trifluoroacetic acid; TIS, triisopropylsilane;

3. Synthesis of building blocks

Route 2 (f,g,h) was chosen for the synthesis of Fmoc-L-Phe-CCH given the minimal racemization observed following this synthetic path. The rest of the amino alkynes were synthesized following route 1 (c,d,e).

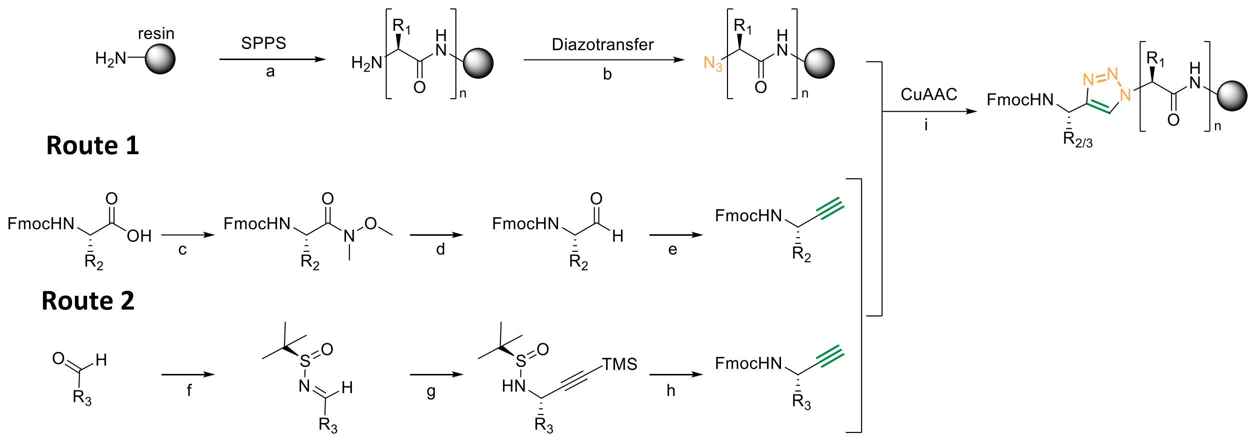

Scheme S1: R_1 refers to amino acid specific side chain. R_2 corresponds to the protected side chains of Asn, DTrp, Thr and R_3 corresponds to the side chain of Phe. a) i) amino acids, HATU, DIPEA ii) piperidine; b) ISA·HCl, DIPEA; **Route 1** c) N,O-dimethylhydroxylamine hydrochloride salt, BOP, DIPEA; d) DIBAL-H; e) Bestmann-Ohira reagent, K_2CO_3, MeOH; **Route 2** f) (S)-(-)-tert-butylsulfinamide, $CuSO_4$; g) BuLi, Ethynyltrimethylsilane, $AlMe_3$; h) i) TBAF ii) HCl iii) DIPEA, FmocOSu; i) tetrakis(acetonitrile)Cu(I) hexafluorophosphate, DIPEA, TBTA.

Route 1[1]

3.1 Synthesis of Weinreb amides

BOP (1 equiv.), DIPEA (2.5 equiv.) and *N*,*O*-dimethylhydroxylamine hydrochloride (1.2 equiv.) were added to a solution of the corresponding Fmoc-protected amino acid (1 equiv.) in DCM (0.1 M). The solution was stirred overnight at room temperature (RT). Completion of the reaction was determined by TLC. The solvent was evaporated under reduced pressure and the crude was purified *via* flash chromatography.

3.2 Synthesis of α-amino alkynes

The Fmoc-protected Weinreb amide (1.0 equiv.) was dissolved in anhydrous DCM (0.1 M) in an oven dried flask under argon atmosphere and the solution was cooled to -78°C (acetone/dry ice bath). DIBAL-H (3.0 equiv.) was dropwise added under inert atmosphere and the mixture was stirred at -78°C until the reaction was completed (typically 2 h). The remaining DIBAL-H was quenched by addition of MeOH (1 mL) and the reaction mixture was allowed to warm to 0°C (ice/ water bath). Additional MeOH (1 mL) was added along with K_2CO_3 (3.0 equiv.) and the Bestmann-Ohira reagent (2.0 equiv.). The reaction stirred at RT overnight. After the addition of DCM and an aqueous solution of 0.5% sodium tartrate the reaction mixture was stirred vigorously. Once both phases became clear, the organic phase was separated, washed with brine, dried over anhydrous Na_2SO_4 and concentrated under reduced pressure. Two different approaches were taken depending on the result obtained at this point. If no Fmoc deprotection was observed the crude was purified by flash chromatography.

In case deprotection of Fmoc group was observed, the crude was dissolved in DCM (0.1 M) and DIPEA (2.5 equiv.) and Fmoc-OSu (2 equiv.) were added, and the reaction was stirred at RT overnight. After completion, the solvent was removed under reduced pressure and the crude was purified via flash chromatography.

Route 2[2]

3.3 Synthesis of N-sulfinyl imine

(S)-(-)-*tert*-butylsulfinamide (1.0 equiv.) was dissolved in DCM (1M) and the freshly distilled phenylacetaldehyde (1.2 equiv.) was added followed by $CuSO_4$ (1.5 equiv.). The reaction was stirred at RT for 72 h and the conversion to the corresponding sulfinimine was checked by TLC. After completion, an aqueous solution of $KHSO_4$ (5%) was added to the suspension. The aqueous layer was extracted with DCM, the combined organic phases were dried over anhydrous Na_2SO_4 and the solvent was removed under reduced pressure. The crude was purified by flash chromatography.

3.4 Synthesis of N-sulfinyl propargylamines

A solution of *n*-BuLi (1.6 M in anhydrous hexane, 1.6 equiv.) was dropwise added at -78°C (acetone/ dry ice bath) to an ethynyltrimethylsilan solution (0.85 M in anhydrous THF, 1.5 equiv.) in an ovendried flask under argon atmosphere. The reaction was stirred at -78°C for two h. Then, a 0.1 M solution of the N-sulfinyl imine (1.0 equiv.) and $AlMe_3$ (0.5 equiv.) was prepared in anhydrous toluene and dropwise added to the first reaction mixture. Following completion (typically 2 h), verified by TLC, the reaction was let warm up to RT and diluted with an aqueous solution of $KHSO_4$ (5%). The mixture was washed with one additional portion of $KHSO_4$ (5%) and the combined aqueous layers were extracted with Et_2O. The combined organic layers were dried over Na_2SO_4 and the crude mixture was concentrated under reduced pressure. The crude product was used in the following step without further purification.

3.5 Desilylation of N-sulfinyl propargylamines

The TMS protected *N*-sulfinyl propargylamine (1.0 equiv.) was dissolved in THF (final concentration of 0.2 M) and TBAF (2.0 equiv.) solution in THF (1 M) was dropwise added at 0 °C. The reaction was stirred for 2 h at 0 °C and 2 h at RT. The mixture was quenched with saturated aqueous solution of NH_4Cl. The organic layer was separated, and the aqueous layer was extracted with Et_2O. The organic layers were combined, dried over anhydrous Na_2SO_4 and concentrated in vacuo. The product was used without further purification.

3.6 Sulfinamide cleavage and Fmoc protection

The N-sulfinyl propargylamine was dissolved in MeOH (0.1 M) and a 4M solution of HCl (3.0 equiv) in dioxane was dropwise added while stirring. The deprotection was verified by TLC (typically 30 min) and the reaction mixture was concentrated under reduced pressure. The residue was dissolved in DCM (0.1 M) and DIPEA (2.5 equiv.) and Fmoc-OSu (2 equiv.) were added, and the reaction was let stir at RT overnight. After completion, the solvent was removed in vacuo and the crude was purified via flash chromatography.

4. Characterization of α-amino alkynes

4.1 Fmoc-L-Phe-CCH

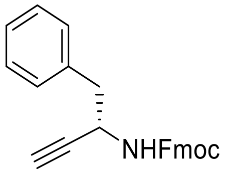

^{1}H NMR (600 MHz, $CDCl_3$) δ 7.77 (d, ^{3}J = 7.5 Hz, 2H), 7.57 (dd, ^{3}J = 5.6, 5.6 Hz, 2H), 7.41 (dd, ^{3}J = 7.5, 7.5 Hz, 2H), 7.33 – 7.25 (m, 7H), 4.99 – 4.91 (m, 1H), 4.80 – 4.72 (m, 1H), 4.48 (d, J = 8.9 Hz, 1H), 4.47 – 4.27 (m, 1H), 4.21 (t, ^{3}J = 6.7 Hz, 1H), 3.21 – 2.80 (m, 2H), 2.32 (d, ^{4}J = 2.2 Hz, 1H).

^{13}C NMR (151 MHz, $CDCl_3$) δ 155.3, 144.0, 143.9, 141.5, 136.1, 13007, 128.5, 127.9, 127.2, 125.2, 125.1, 120.2, 77.2, 72.8, 67.0, 47.3, 44.4, 41.6.

ESI-HRMS: [M+Na]$^{+}$ *m/z* calculated for $C_{25}H_{21}NNaO_2$: 390.1464, measured: 390.1464

The analytical data was found identical to the one reported in literature.

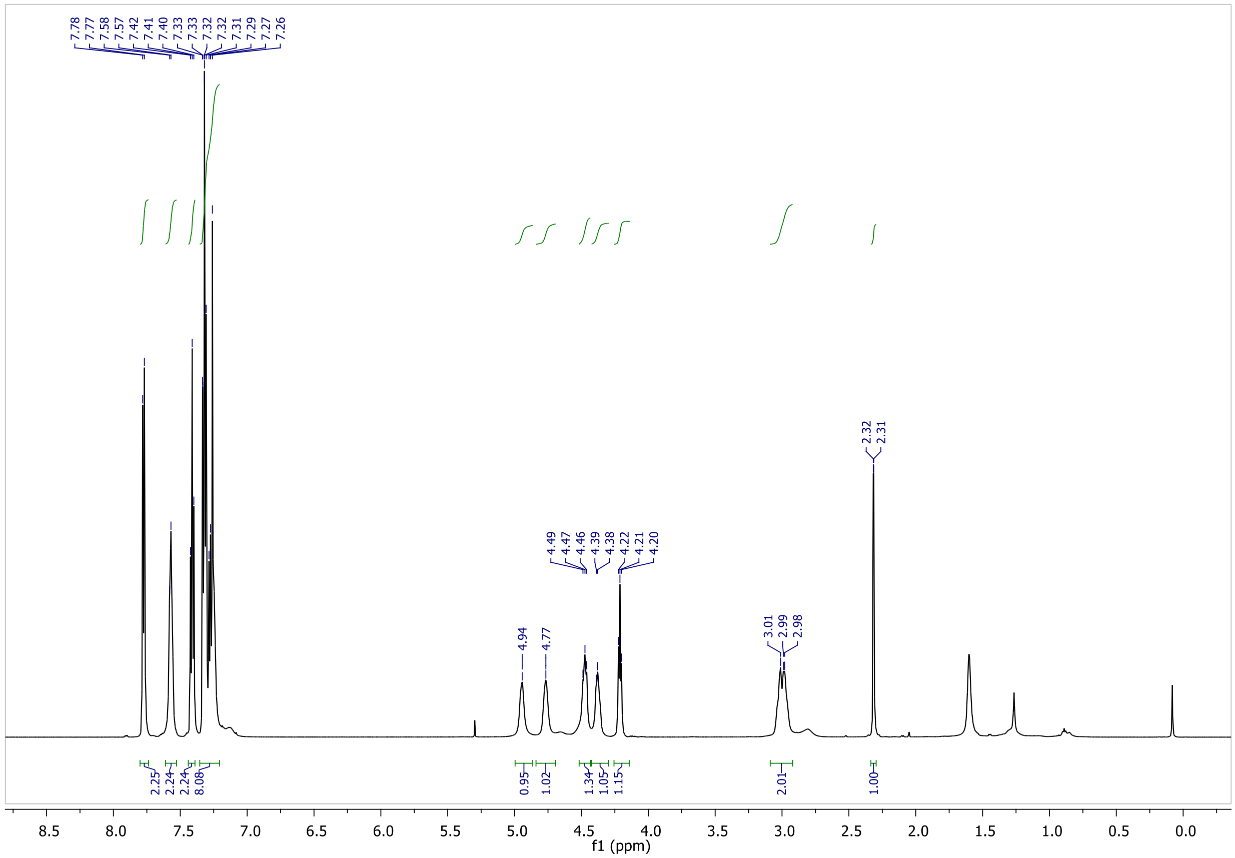

Figure S1: ^{1}H NMR of Fmoc-L-Phe-CCH ($CDCl_3$).

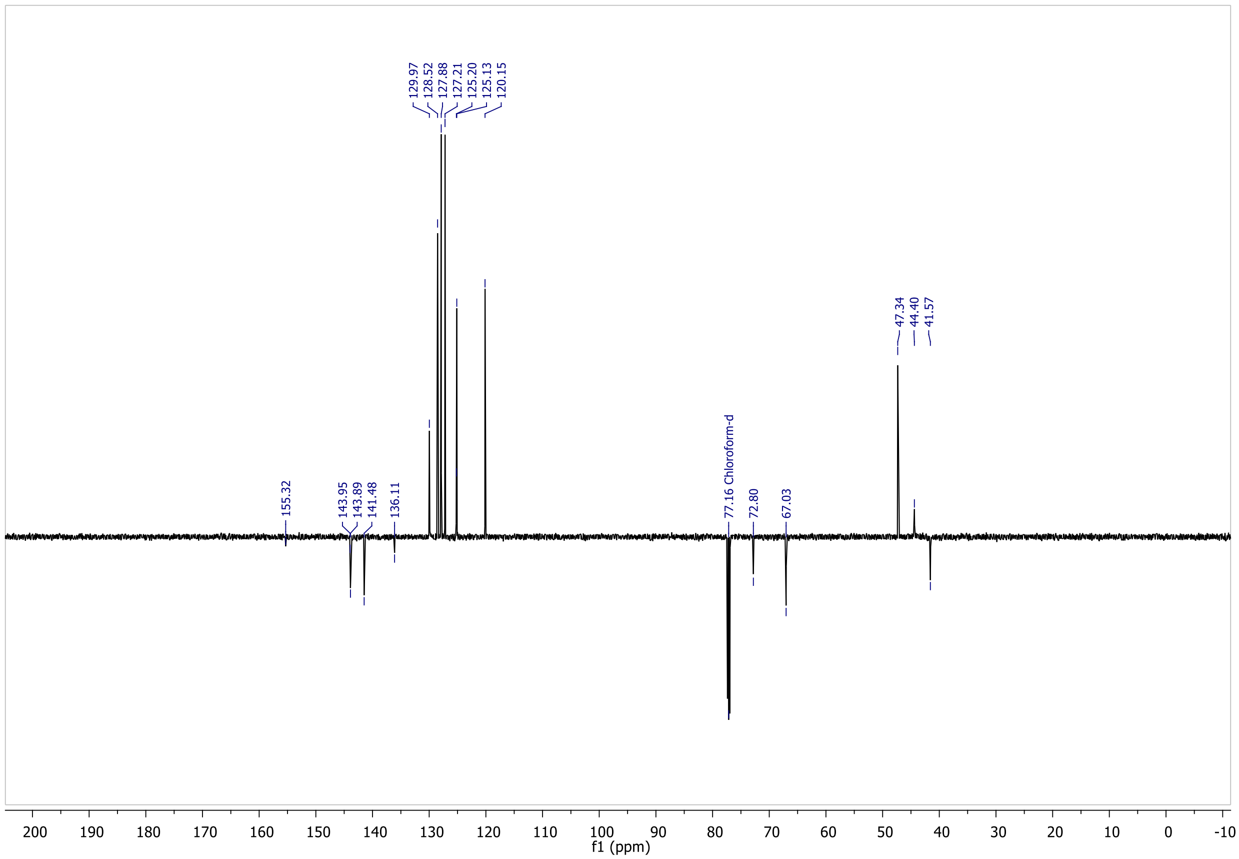

Figure S2: ^{13}C NMR of Fmoc-L-Phe-CCH (qDEPT-135, $CDCl_3$).

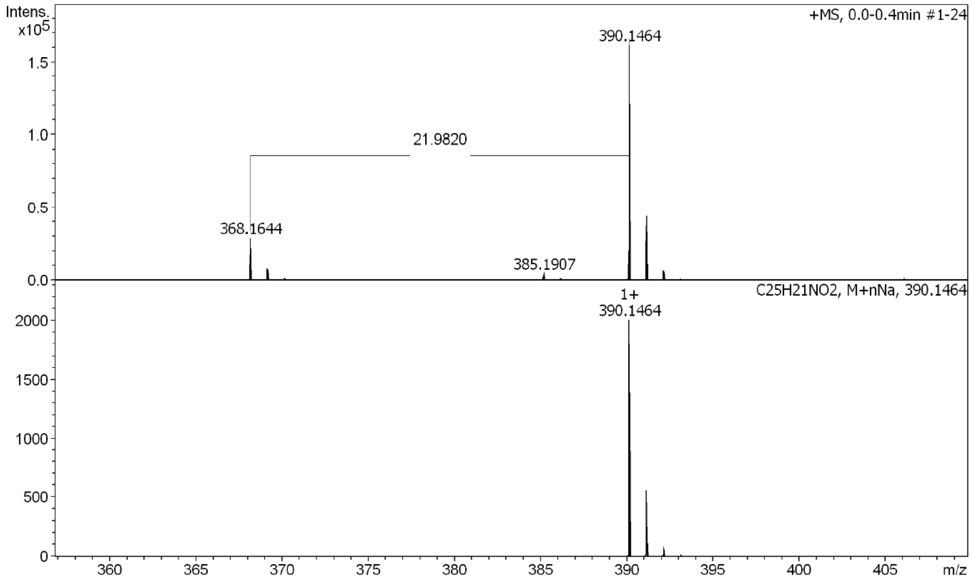

Figure S3: HRMS analysis of Fmoc-L-Phe-CCH (ESI-MS).

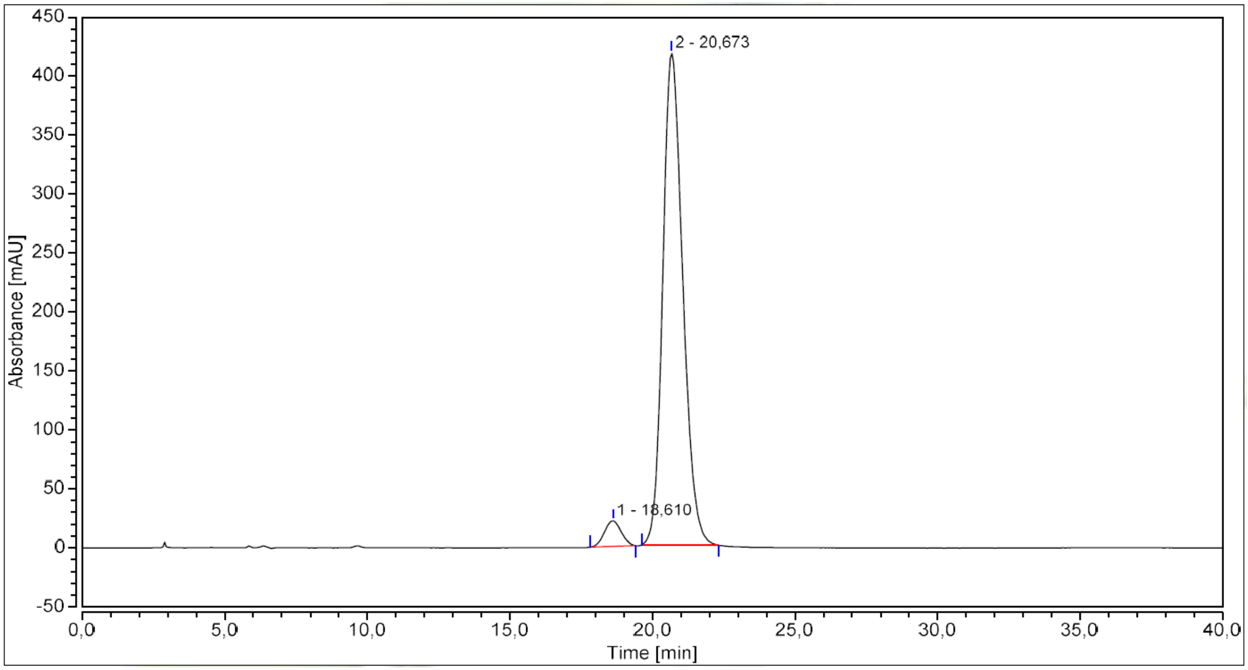

Figure S4: Chiral-HPLC analysis of Fmoc-L-Phe-CCH showing partial racemization. Fmoc-D-Phe-CCH (Peak 1, r_t=18.610 min, 5%) and Fmoc-L-Phe-CCH (Peak 2, r_t = 20.673 min, 95%). Isocratic elution in 20% isopropanol (IPA) in Hexane.

4.2 Fmoc-L-Thr(tBu)-CCH

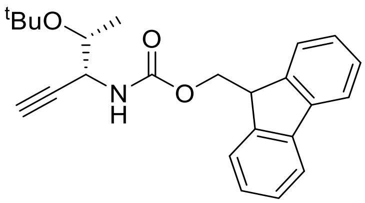

^{1}H NMR (600 MHz, $CDCl_3$) δ 7.77 (d, J = 7.5 Hz, 2H), 7.61 (d, J = 7.0 Hz, 2H), 7.41 (dd, J = 7.4, 7.4 Hz, 2H), 7.32 (td, J = 7.5, 0.8 Hz, 2H), 5.17 (s, 1H), 4.46 – 4.38 (m, 2H), 4.24 (t, $_{3}J$ = 6.5 Hz, 1H), 3.84 (s, 1H), 2.28 (d, $_{3}J$ = 2.4 Hz, 1H), 1.24 (s, 9H), 1.19 (d, $_{4}J$ = 6.2 Hz, 4H).

^{13}C NMR (151 MHz, $CDCl_3$) δ 155.9, 144.0, 143.9, 141.5, 127.8, 127.2, 125.2, 120.1, 82.1, 77.4, 77.2, 77.1, 76.9, 74.3, 72.1, 68.4, 67.1, 53.6, 49.3, 47.4, 28.7, 28.5, 28.4, 19.8, 19.4.

ESI-HRMS: $[M+H]^+$ m/z calculated for $C_{24}H_{27}NO_3$: 378.2064, measured: 378.2058

The analytical data was found identical to the one reported in literature.

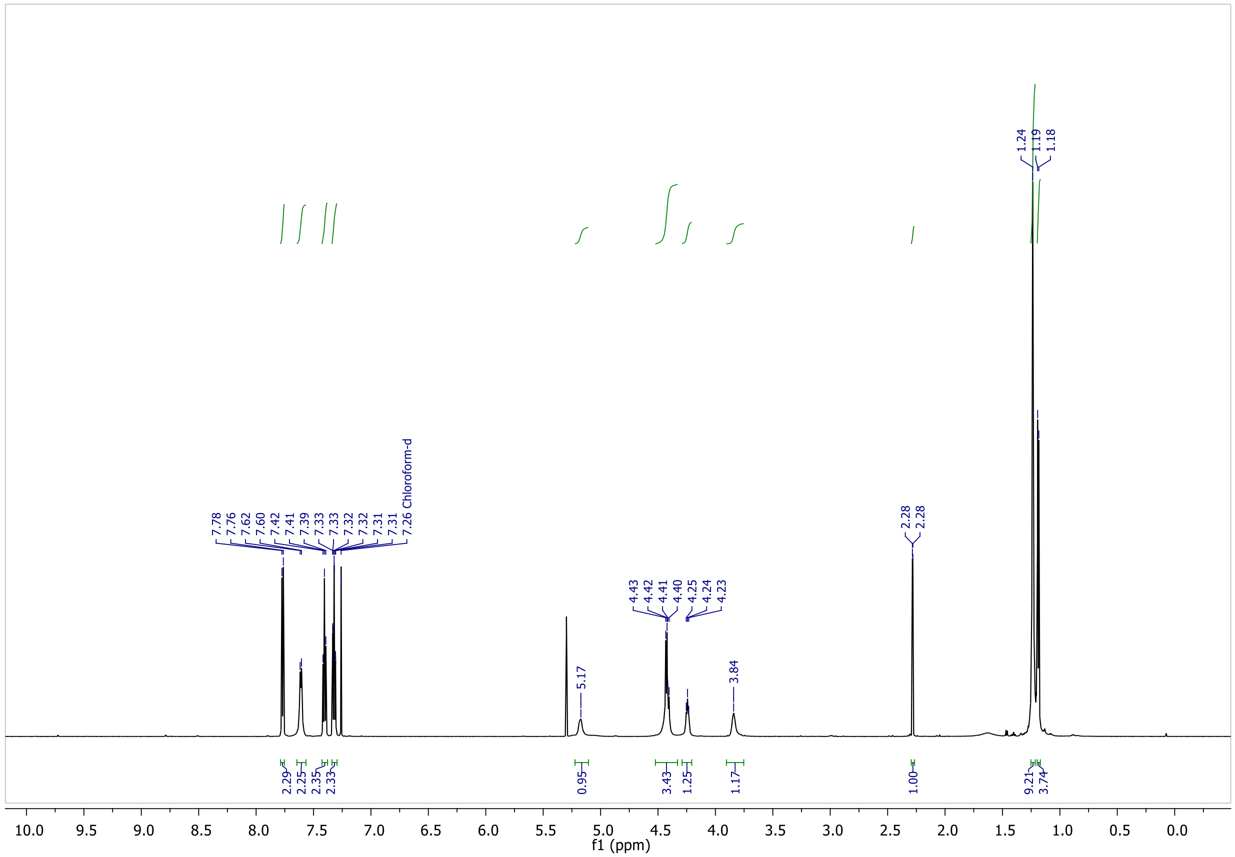

Figure S5: ^{1}H NMR of Fmoc-L-Thr(tBu)-CCH ($CDCl_3$).

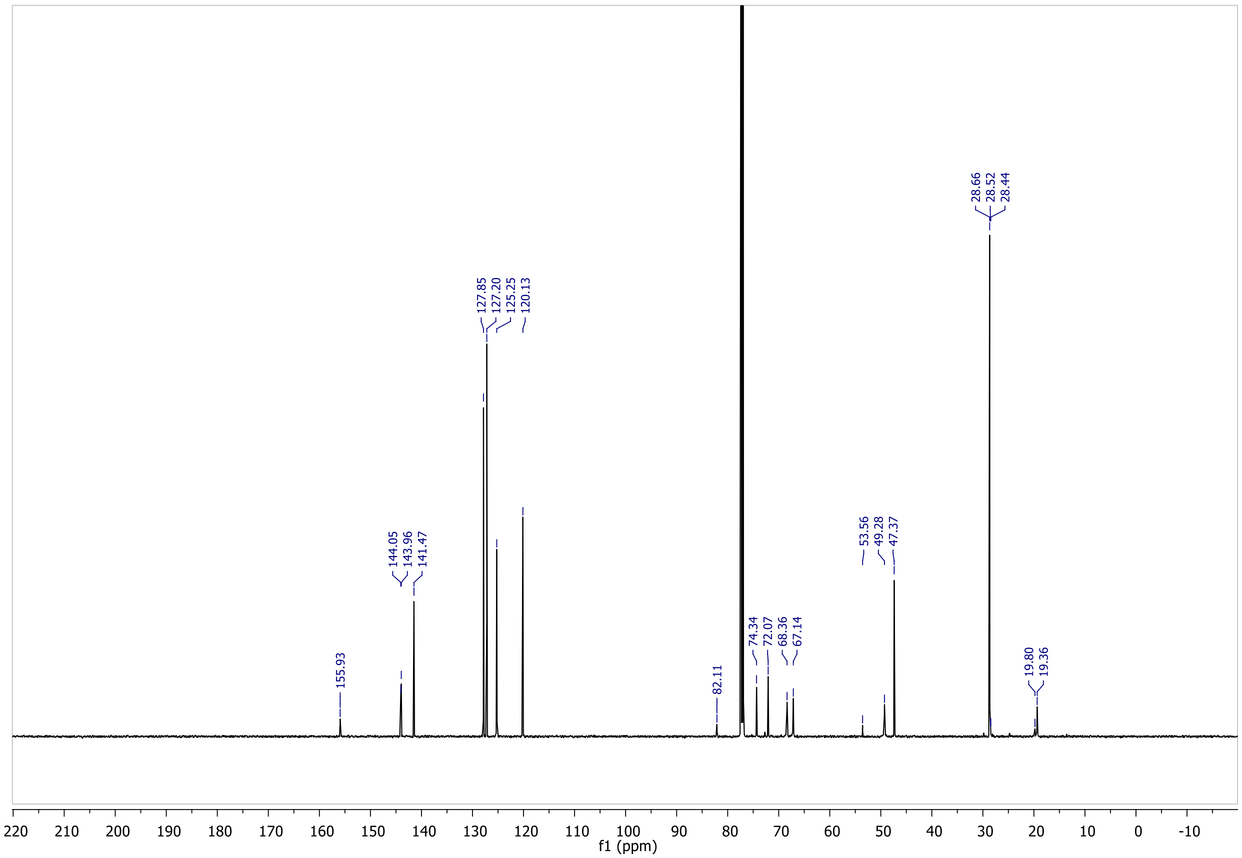

Figure S6: ^{13}C NMR of Fmoc-L-Thr(tBu)-CCH (DEPT, $CDCl_3$).

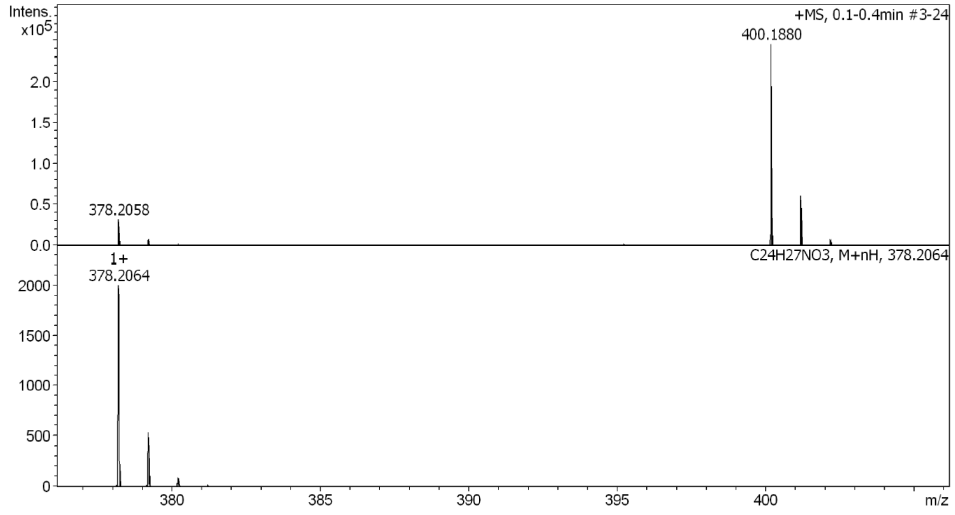

Figure S7: HRMS analysis of Fmoc-L-Thr(tBu)-CCH (ESI-MS).

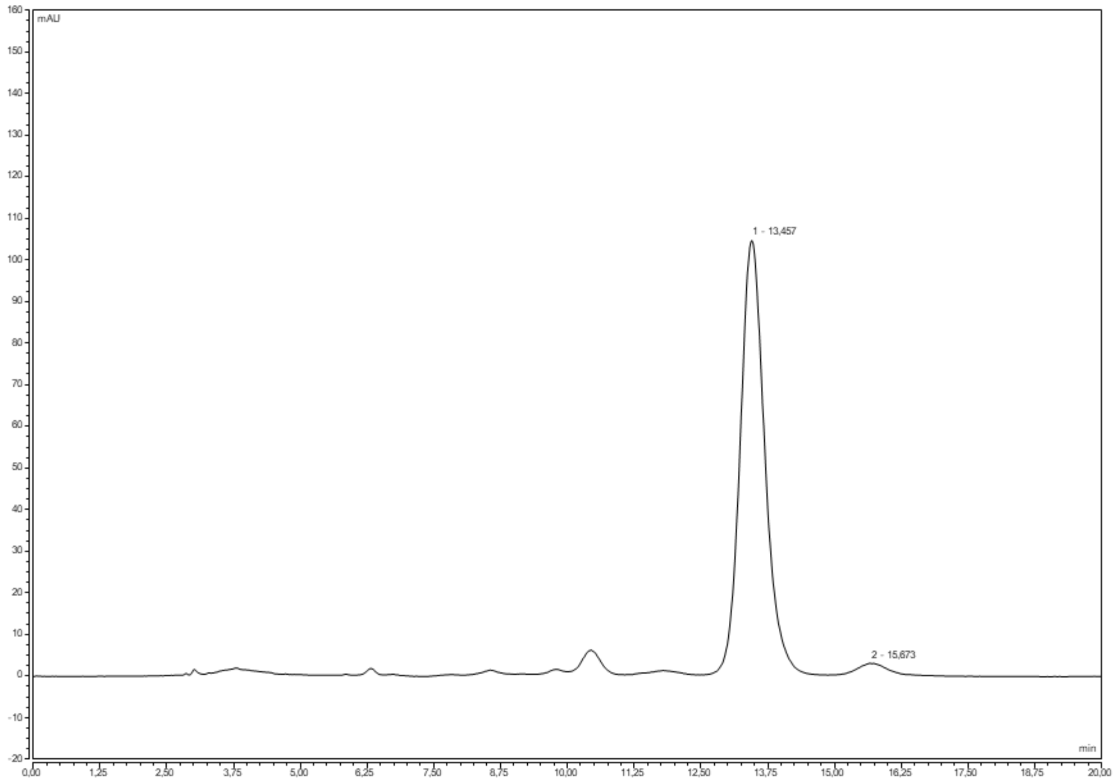

Figure S8: Chiral-HPLC analysis of Fmoc-L-Thr(OtBu)-CCH showing partial racemization. Fmoc-L-Thr(OtBu)-CCH (Peak 1, r_t=13.457 min, 97%) and Fmoc-D-Thr(OtBu)-CCH (Peak 2, r_t=15.673 min, 3%). Isocratic elution in 10% IPA in Hexane.

4.3 Fmoc-D-Trp(Boc)-CCH

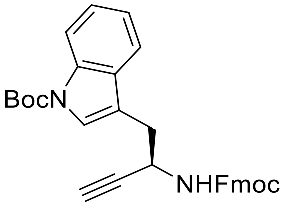

^{1}H NMR (700 MHz, $CDCl_3$) δ 8.14 (s, 1H), 7.77 (d, *J* = 7.3 Hz, 2H), 7.70 – 7.49 (m, 4H), 7.40 (t, *J* = 7.4 Hz, 2H), 7.36 – 7.19 (m, 4H), 5.12 (d, *J* = 7.0 Hz, 1H), 4.87 (s, 1H), 4.42 (dt, *J* = 17.5, 10.2 Hz, 2H), 4.21 (t, *J* = 7.0 Hz, 1H), 3.27 – 3.15 (m, 1H), 3.14 – 2.88 (m, 1H), 2.32 (d, *J* = 2.3 Hz, 1H), 1.67 (s, 9H).

^{13}C NMR (176 MHz, $CDCl_3$) δ 155.5, 149.8, 143.9, 143.9, 141.4, 135.4, 130.8, 127.9, 127.8, 127.2, 127.2, 125.2, 125.2, 124.6, 124.6, 122.7, 120.1, 120.1, 119.2, 115.4, 115.2, 83.8, 82.7, 77.2, 72.6, 67.2, 47.3, 43.7, 31.5, 28.3.

ESI-HRMS: $[M+Na]^+$ *m/z* calculated for $C_{32}H_{30}N_2NaO_4$: 529.2028, measured: 529.2028.

The analytical data was found identical to the one reported in literature.

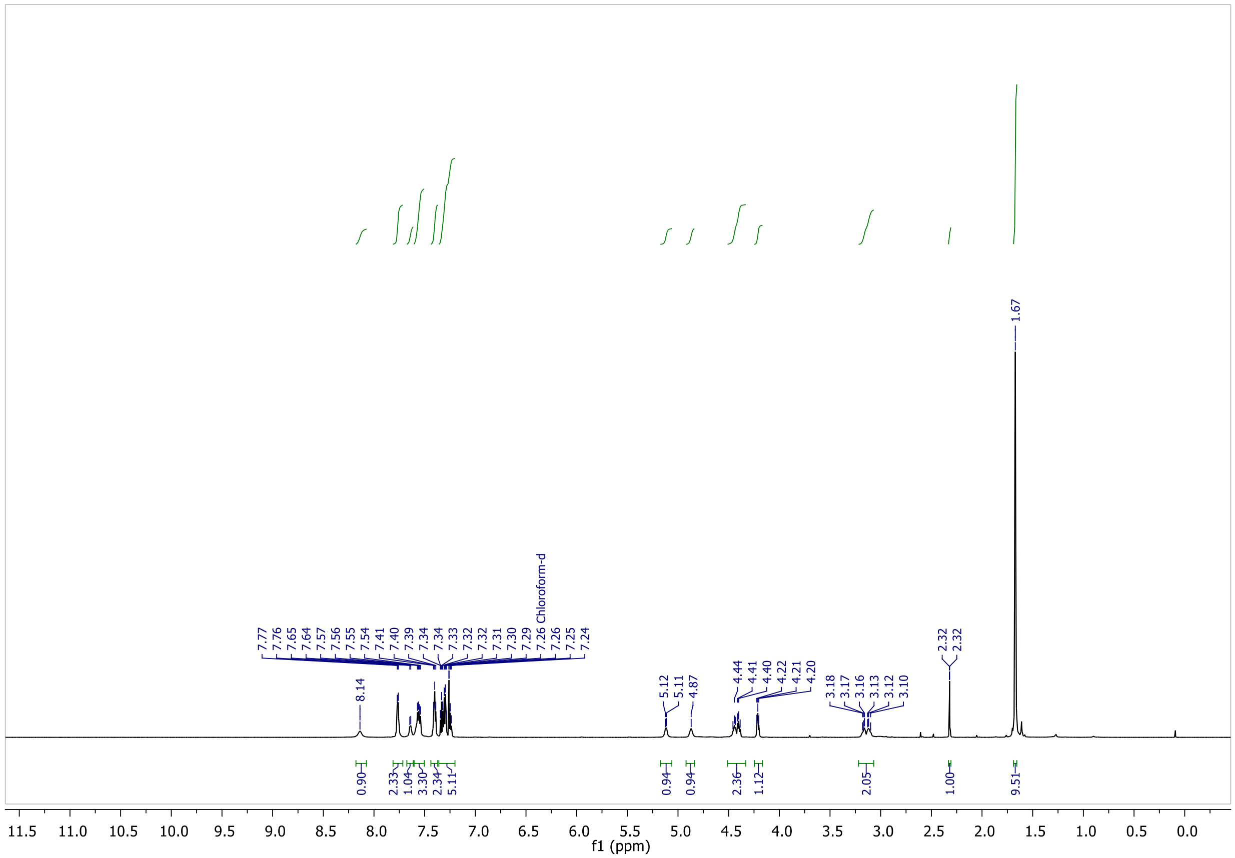

Figure S9: ^{1}H NMR of Fmoc-D-Trp(Boc)-CCH ($CDCl_3$).

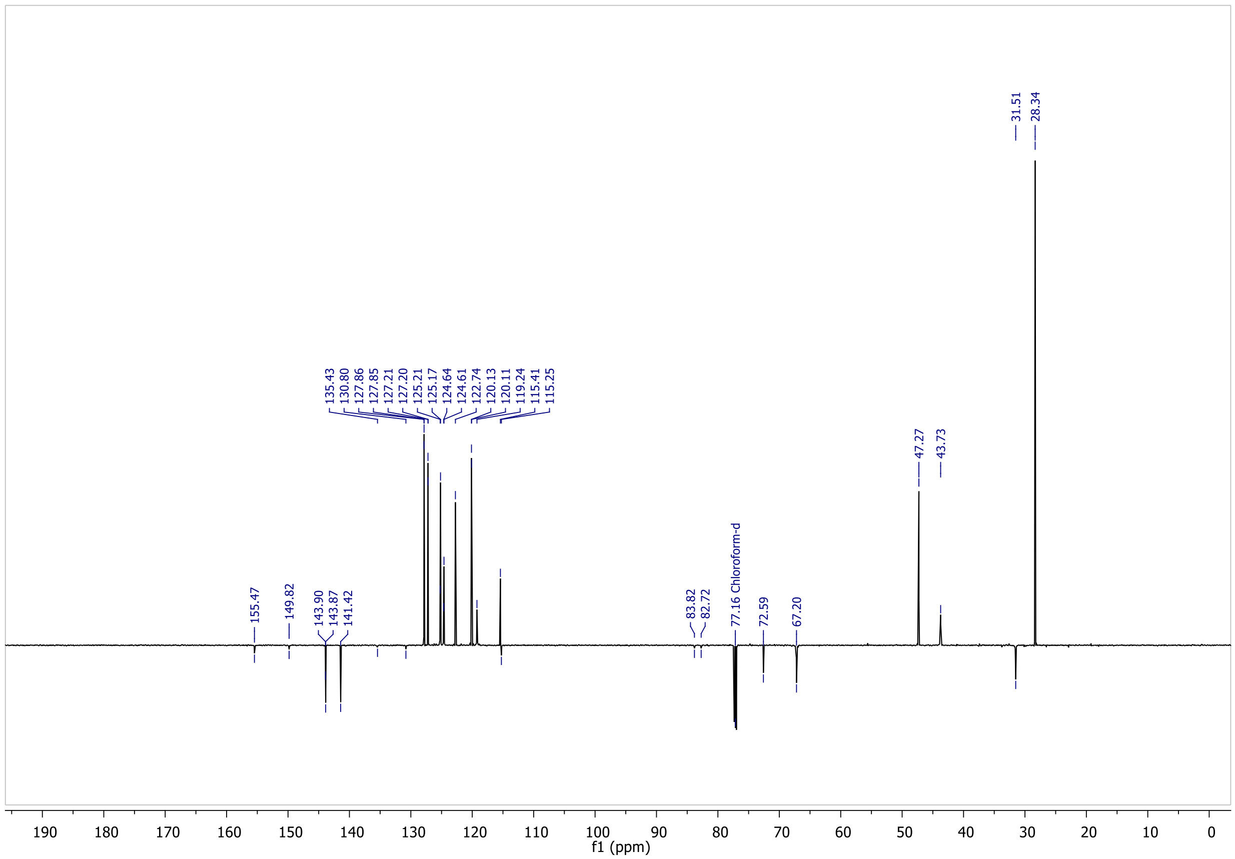

Figure S10: ^{13}C NMR of Fmoc-D-Trp(Boc)-CCH (qDEPT-135, $CDCl_3$).

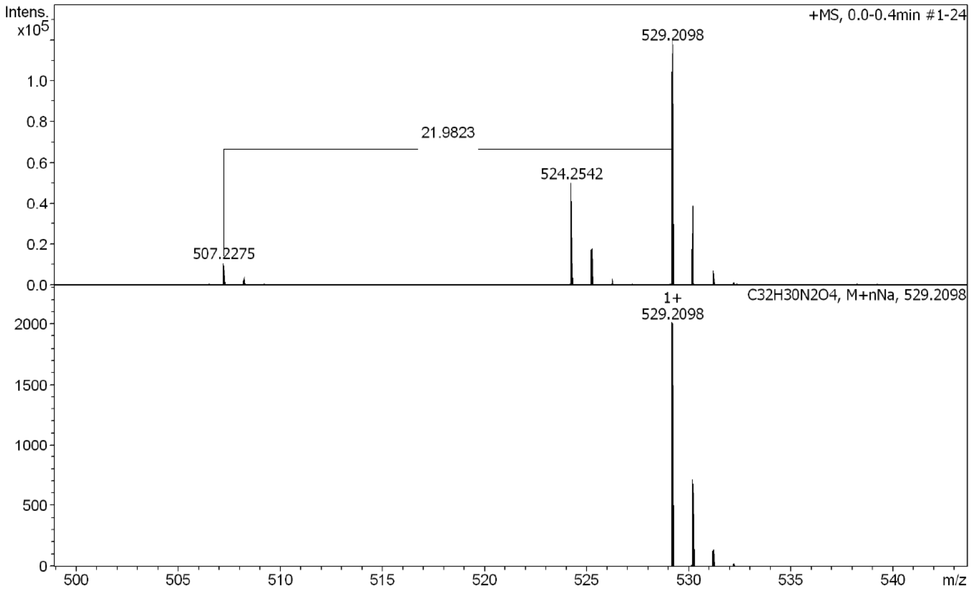

Figure S11: HRMS analysis of Fmoc-D-Trp(Boc)-CCH (ESI-MS).

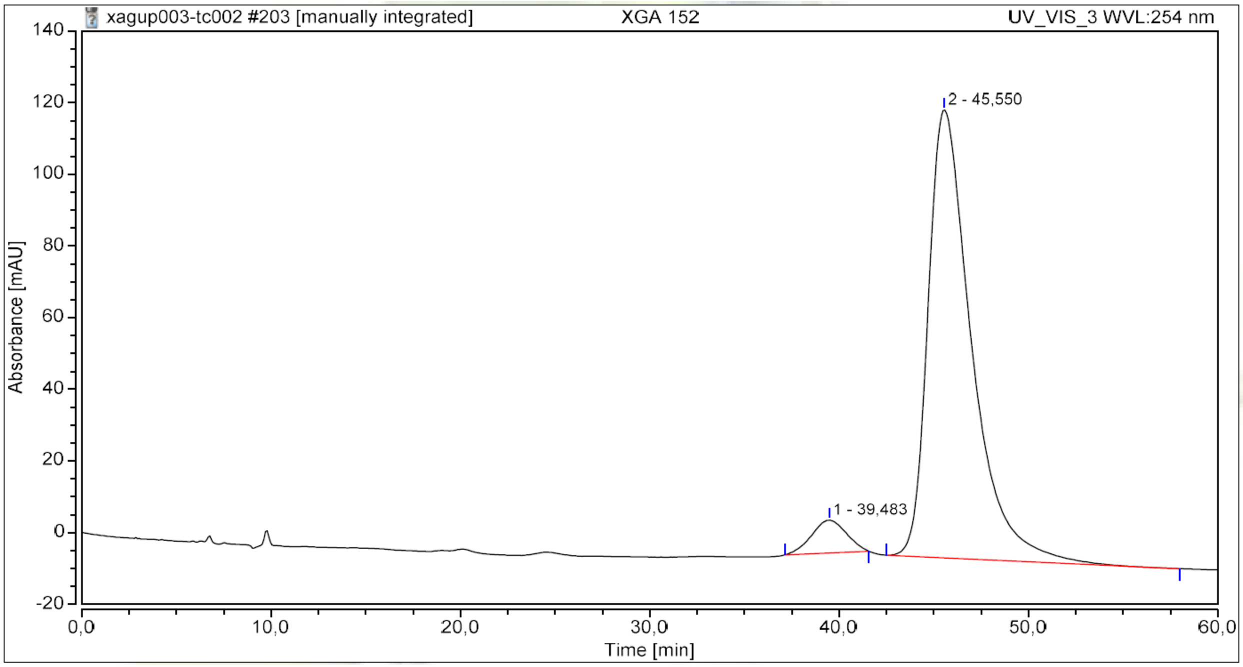

Figure S12: Chiral-HPLC analysis of Fmoc-D-Trp(Boc)-CCH showing partial racemization. Fmoc-L-Trp(Boc)-CCH (Peak 1, r_t = 39.483 min, 7%) and Fmoc-D-Trp(Boc)-CCH (Peak 2, r_t = 45.550 min, 93%). Isocratic elution in 10% IPA in Hexane.

4.4 Fmoc-L-Asn(Trt)-CCH

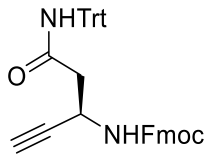

^{1}H NMR (500 MHz, $CDCl_3$) δ 7.75 (d, *J* = 7.5 Hz, 2H), 7.56 (d, *J* = 7.3 Hz, 2H), 7.39 (dd, *J* = 7.4, 7.4 Hz, 2H), 7.35 – 7.19 (m, 20H), 6.80 (s, 1H), 6.27 (d, *J* = 6.5 Hz, 1H), 4.81 (s, 1H), 4.34 (d, *J* = 7.1 Hz, 2H), 4.18 (d, *J* = 6.9 Hz, 1H), 2.73 (s, 2H), 2.37 (s, 1H).

^{13}C NMR (151 MHz, $CDCl_3$) δ 168.9, 144.5, 144.0, 141.4, 128.9, 128.2, 127.8, 127.4, 127.2, 125.3, 120.1, 82.4, 77.2, 72.0, 71.2, 67.3, 47.2, 41.8, 40.7, 29.8.

ESI-HRMS: $[M+Na]^+$ *m/z* calculated for $C_{39}H_{32}N_2NaO_3$: 599.2305, measured: 599.2303

The analytical data was found identical to the one reported in literature.

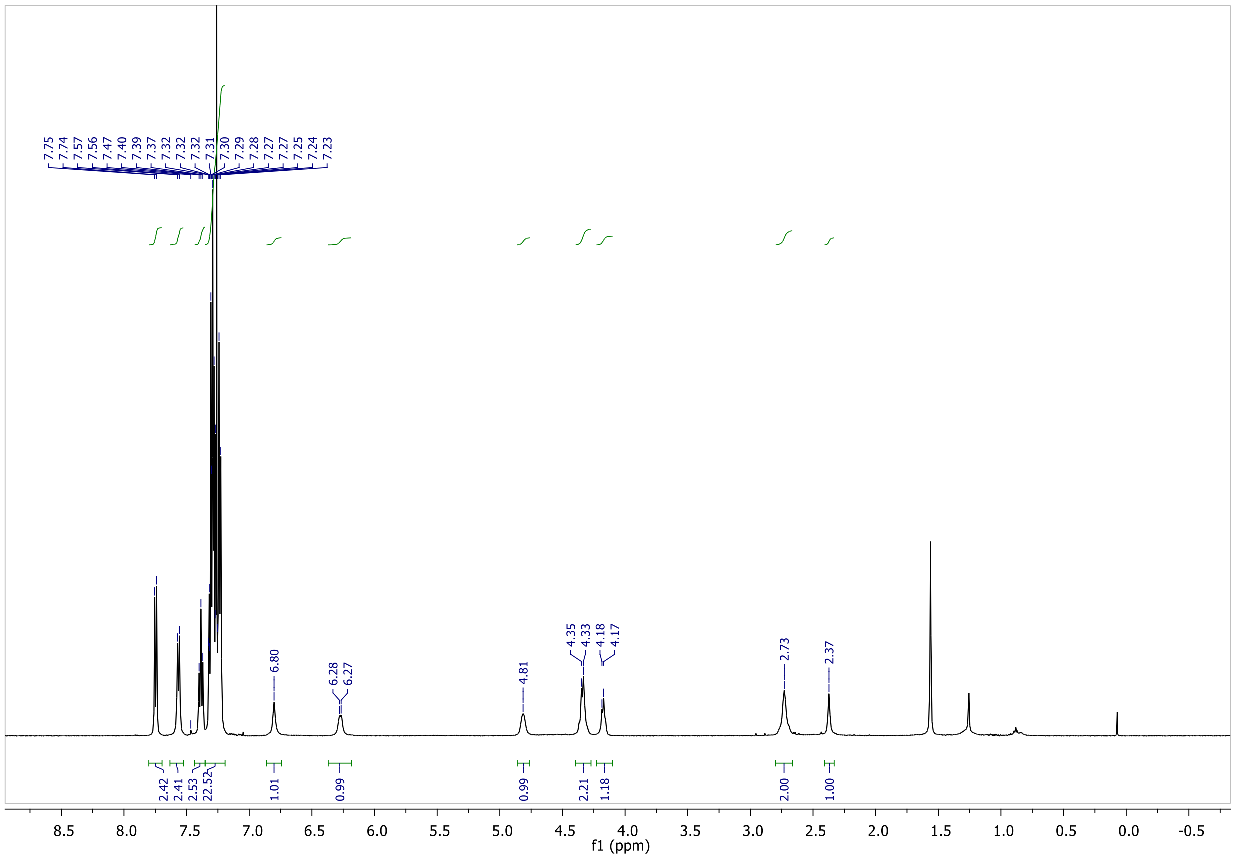

Figure S13: ^{1}H NMR of Fmoc-L-Asn(Trt)-CCH ($CDCl_3$).

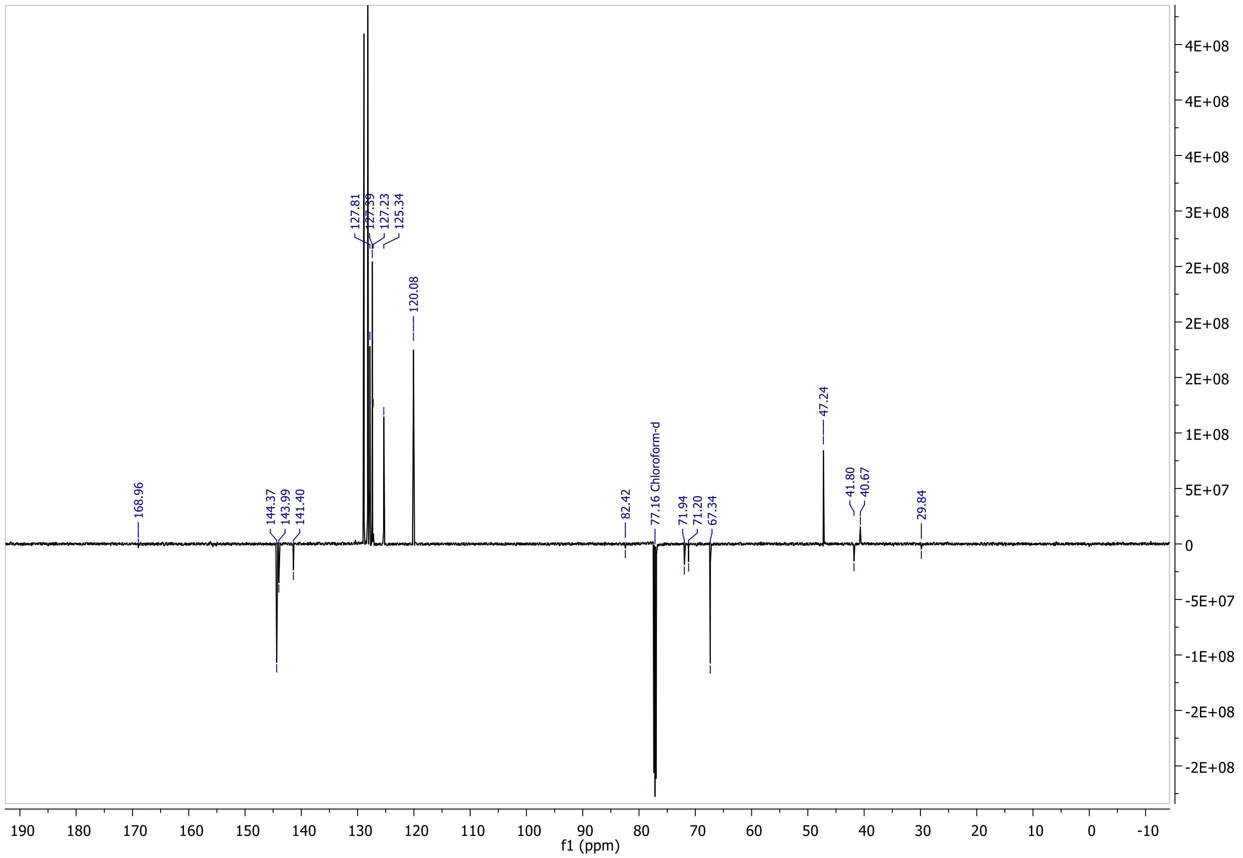

Figure S14: ^{13}C NMR of Fmoc-L-Asn(Trt)-CCH (qDEPT-135, $CDCl_3$).

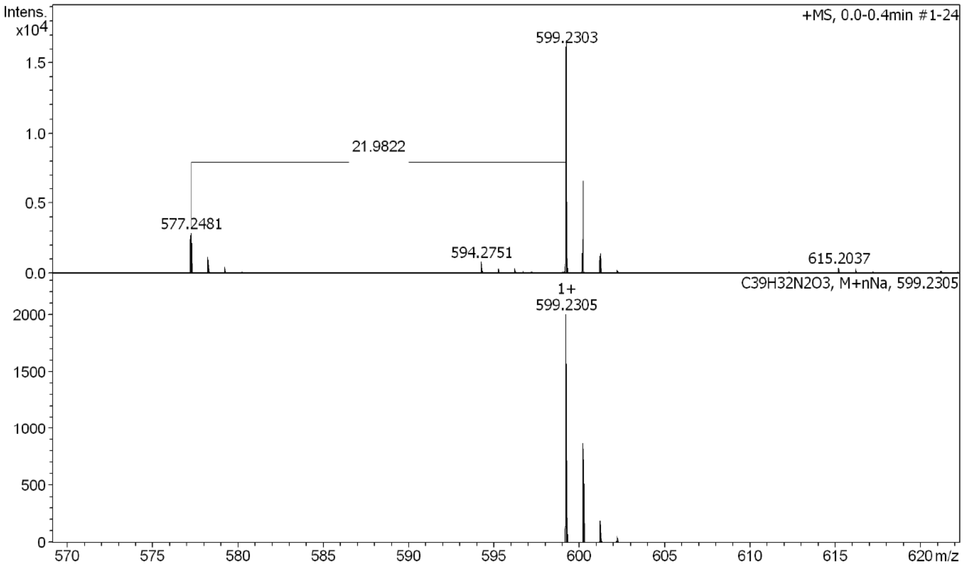

Figure S15: HRMS analysis of Fmoc-L-Asn(Trt)-CCH (ESI-MS).

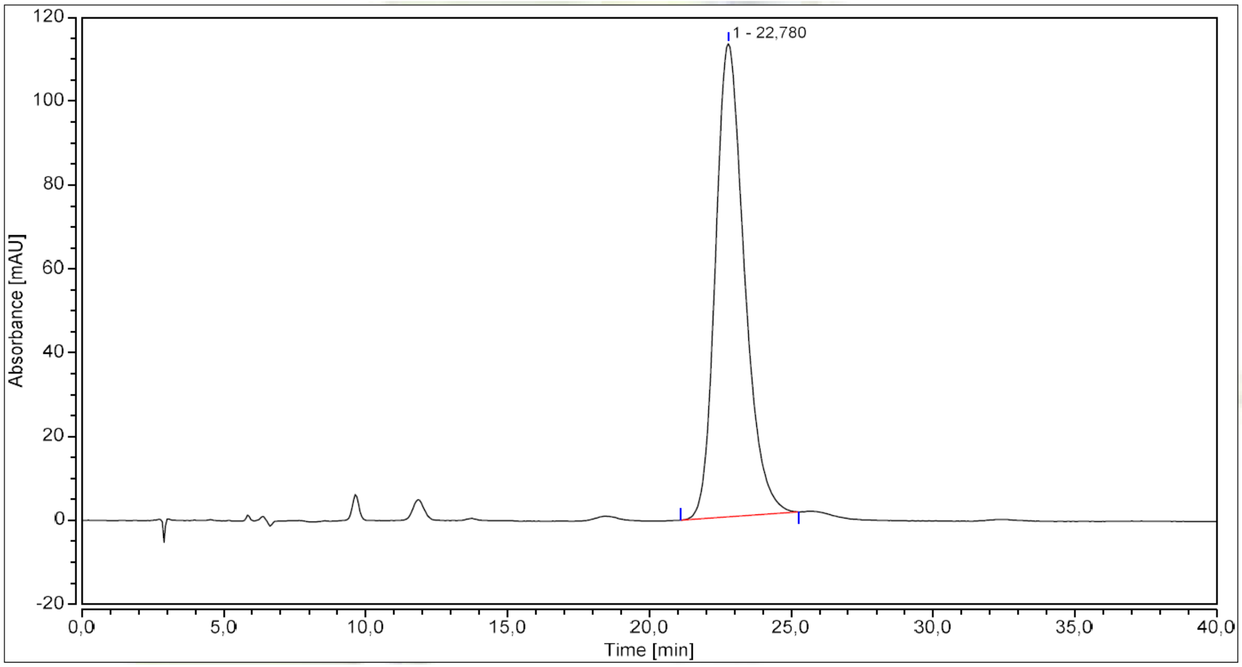

Figure S16: Chiral-HPLC analysis of Fmoc-L-Asn(Trt)-CCH. Fmoc-L-Asn(Trt)-CCH (Peak 1, r_t = 22.780 min, >99%). Isocratic elution in 10% IPA in Hexane.

5. Characterization of triazolo-peptidomimetics

Table S1. HPLC-MS characterization of peptide and triazolo-peptidomimetic conjugates (replica of Table 1 in the main manuscript)

Compound [1]	Structure [2]	Purity [%] [3]	m/z, $[M+2H]^{2+}$ [Da] [4,5]
AT2S	[DOTA, D-Trp^{8}]SST-14	>95	1012.9581
XG1	[DOTA, Asn^{5}-Ψ[Tz]-Phe^{6}, D-Trp^{8}]SST-14	>95	1024.9629
XG2	[DOTA, Phe^{6}-Ψ[Tz]-Phe^{7}, D-Trp^{8}]SST-14	>95	1024.9634
XG3	[DOTA, D-Trp^{8}-Ψ[Tz]-Lys^{9}, D-Trp^{8}]SST-14	>95	1024.9619
XG4	[DOTA, Thr^{10}-Ψ[Tz]-Phe^{11}, D-Trp^{8}]SST-14	>95	1024.9620
XG5	[DOTA, Phe^{11}-Ψ[Tz]-Thr^{12}, D-Trp^{8}]SST-14	>95	1024.9635
^{nat}In-XG1	[^{nat}In-DOTA, Asn^{5}-Ψ[Tz]-Phe^{6}, D-Trp^{8}]SST-14	>95	1080.9044

[1] AT2S is the unmodified all-amide bond reference compound [3] [2] Ψ[Tz] represents the trans-amide bond replaced by a 1,4-disubstituted 1,2,3-triazole [3] Purity was determined by reversed-phase HPLC [4] Molecular masses of peptides were measured by ESI-MS coupled to an HPLC system [5] Expected m/z of AT2S = 1012.9571, XG1-5 = 1024.9627 and ^{nat}In-XG1 = 1080.9035

AT2S

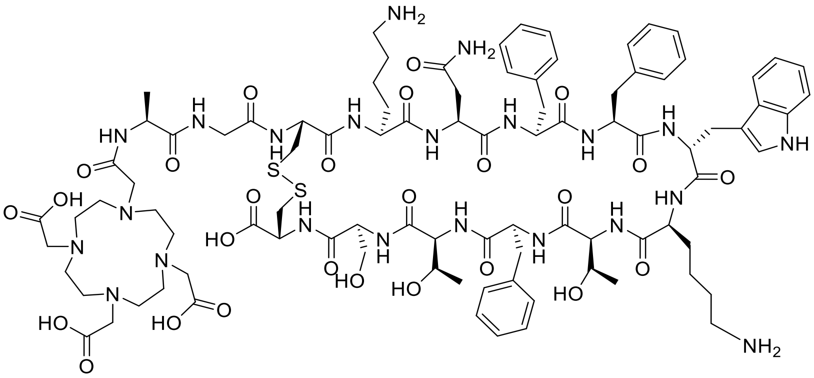

Molecular Weight: 2024,30

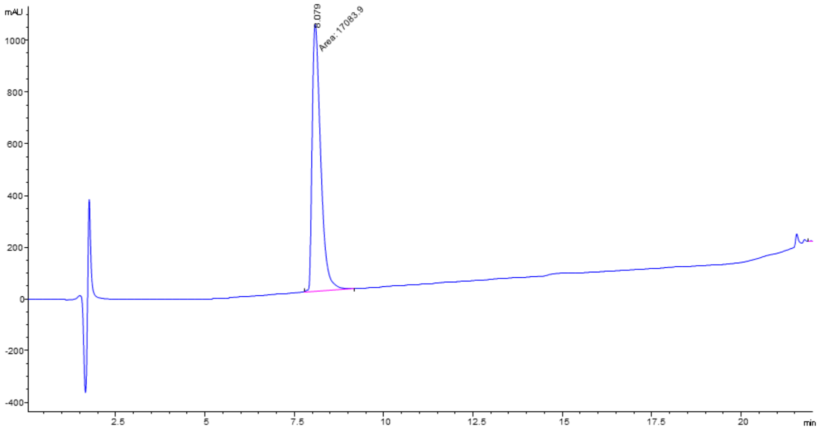

Figure S17: Analytical HPLC chromatogram of purified AT2S, r_t = 8.078 min.

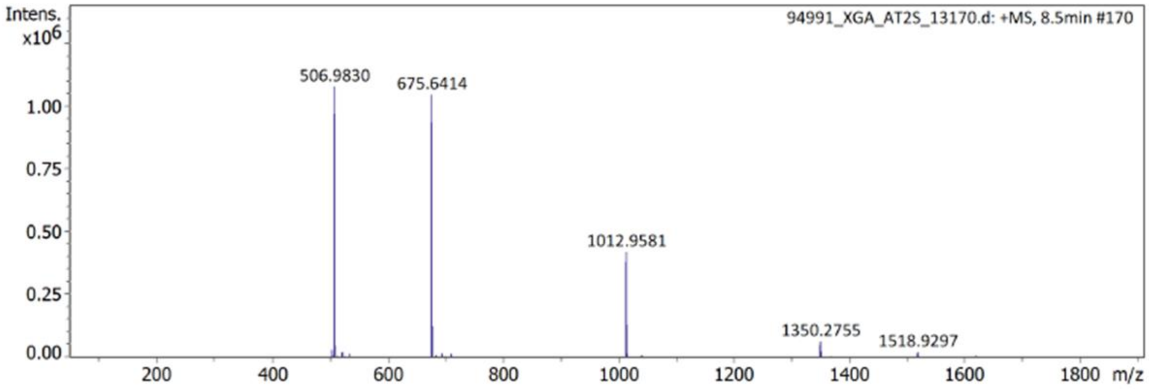

Figure S18: ESI-HRMS of purified AT2S.

XG1

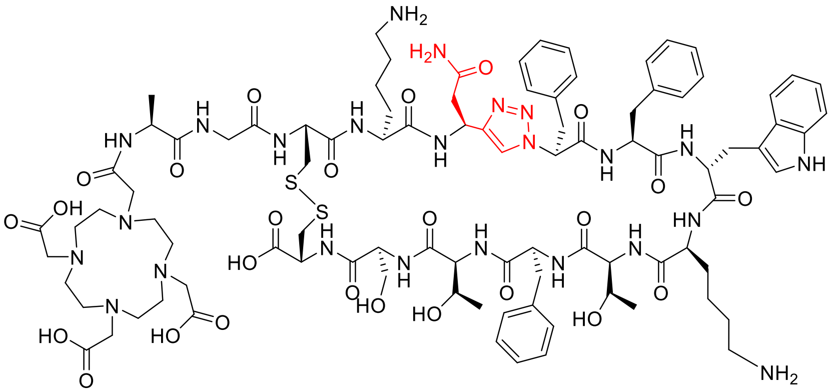

Molecular Weight: 2048,33

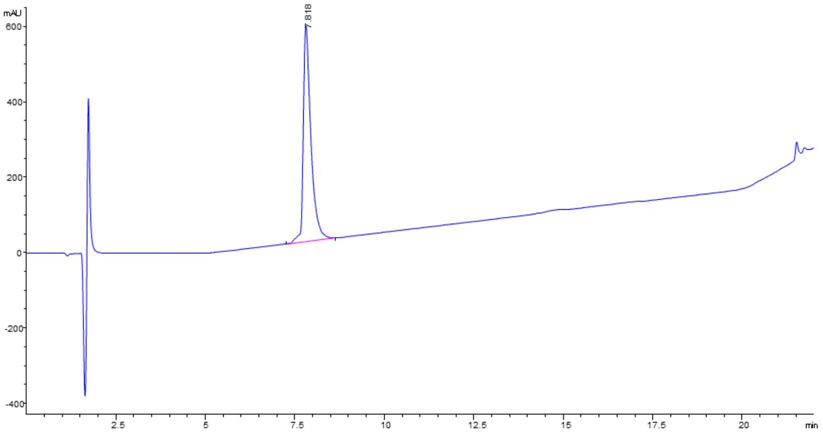

Figure S19: Analytical HPLC chromatogram of purified XG1, r_t = 7.818 min.

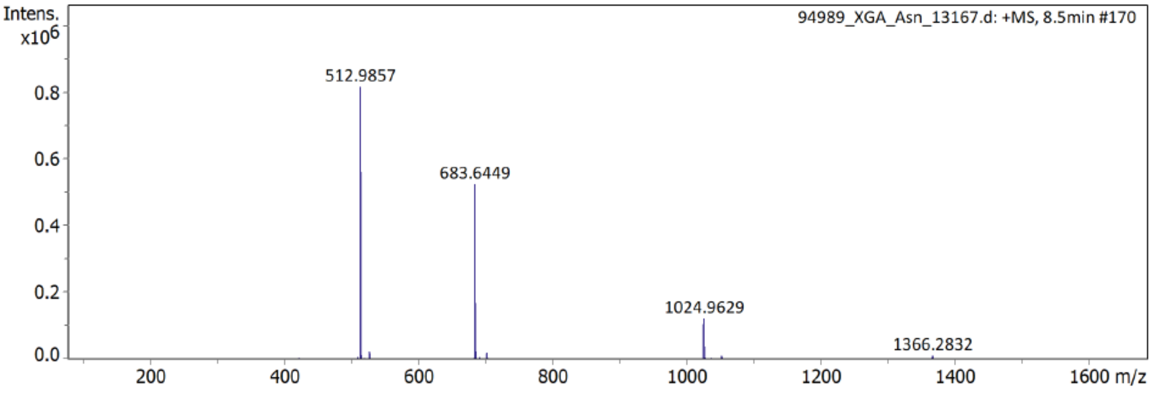

Figure S20: ESI-HRMS of purified XG1.

XG2

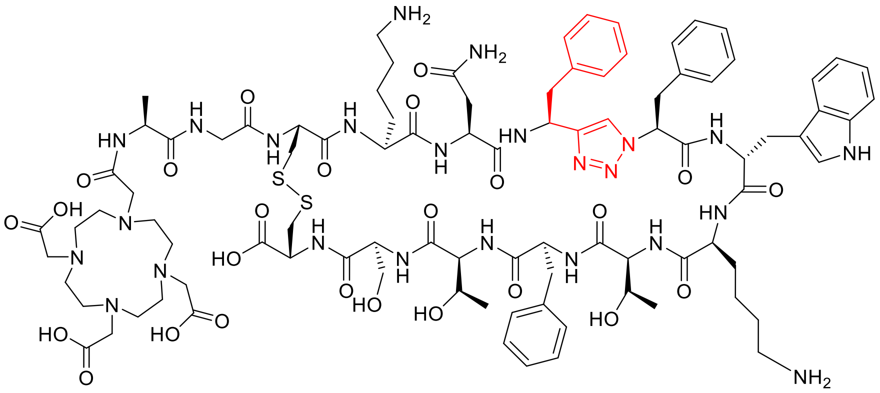

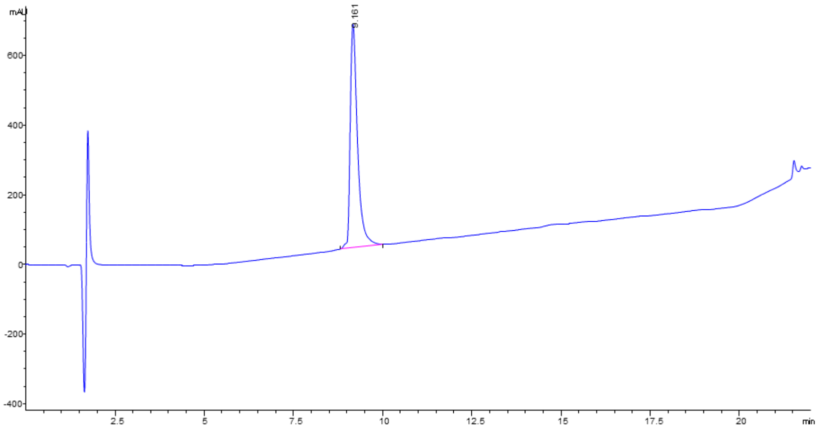

Figure S21: Analytical HPLC chromatogram of purified XG2, r_t = 9.161 min.

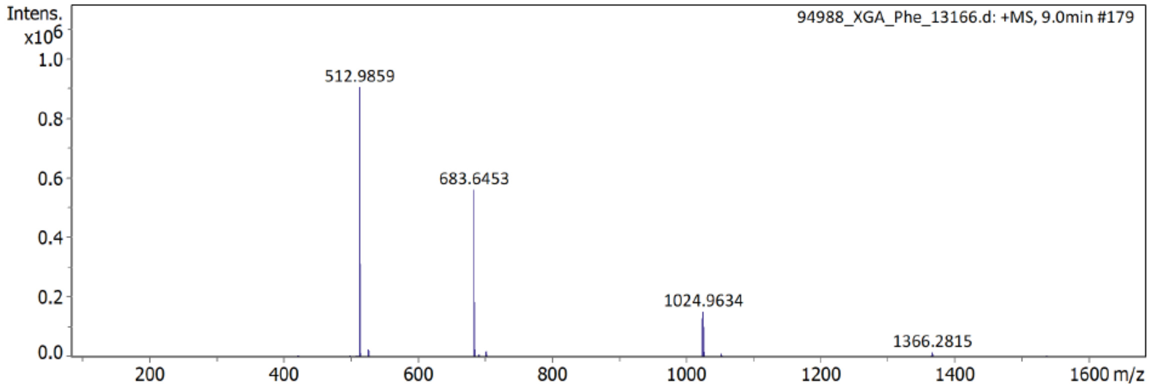

Figure S22: ESI-HRMS of purified XG2.

XG3

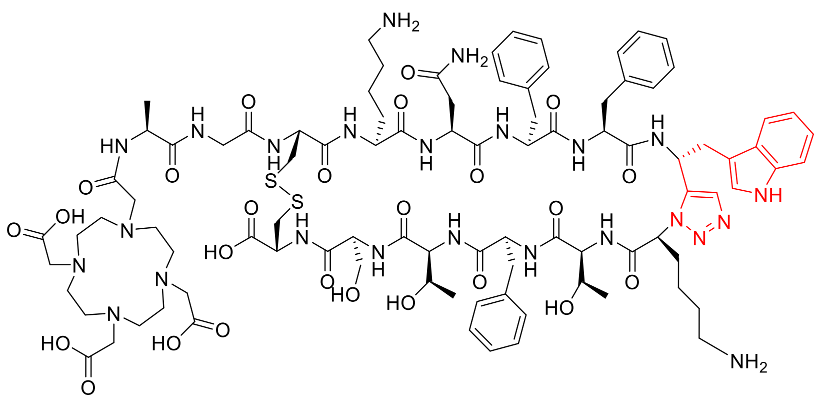

Molecular Weight: 2048,33

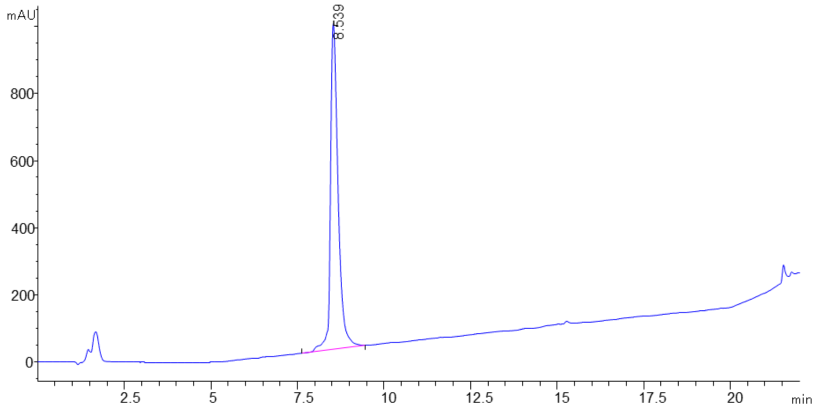

Figure S23: Analytical HPLC chromatogram of purified XG3, r_t = 8.539 min.

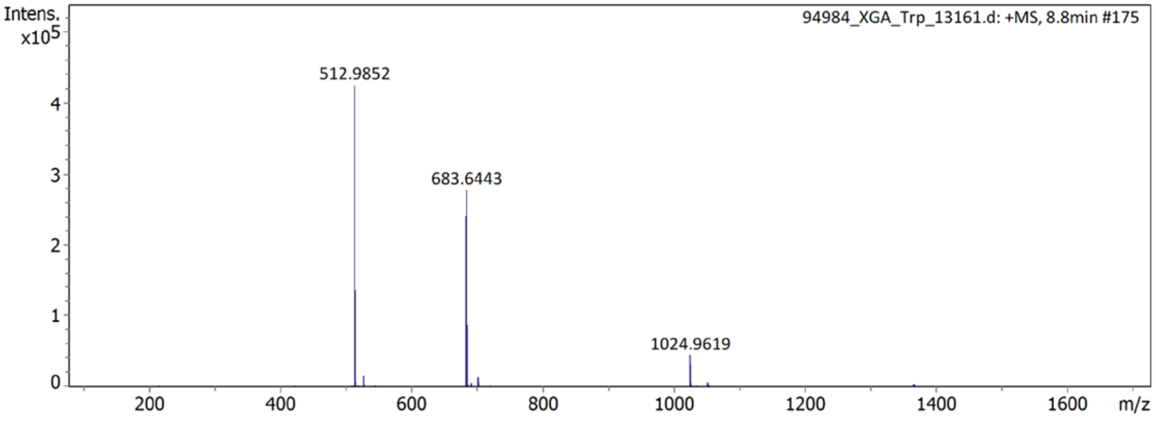

Figure S24: ESI-HRMS of purified XG3.

XG4

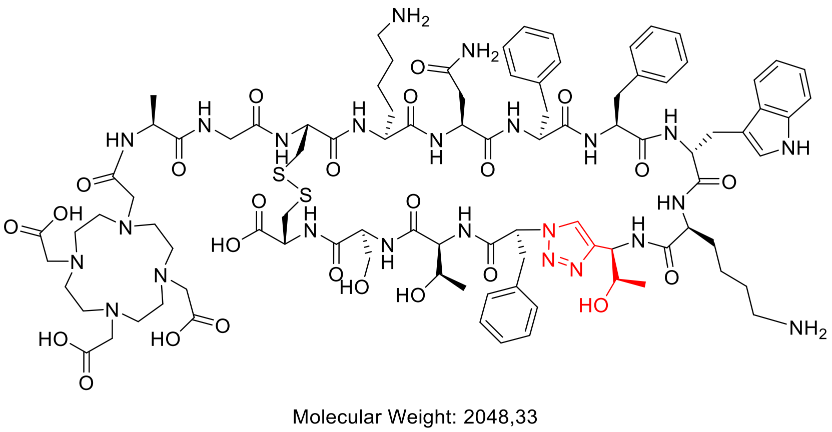

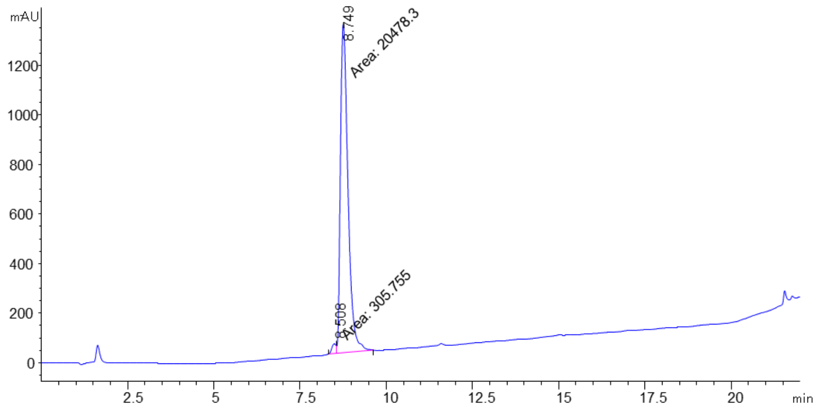

Figure S25: Analytical HPLC chromatogram of purified XG4, r_t = 8.749 min.

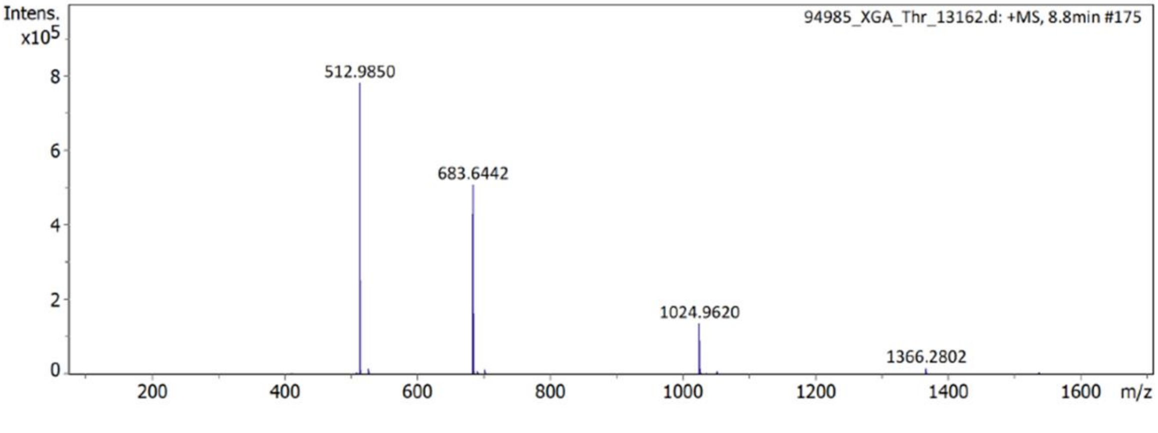

Figure S26: ESI-HRMS of purified XG4.

XG5

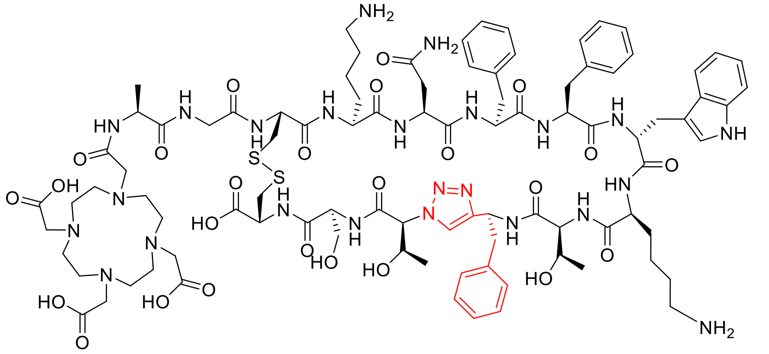

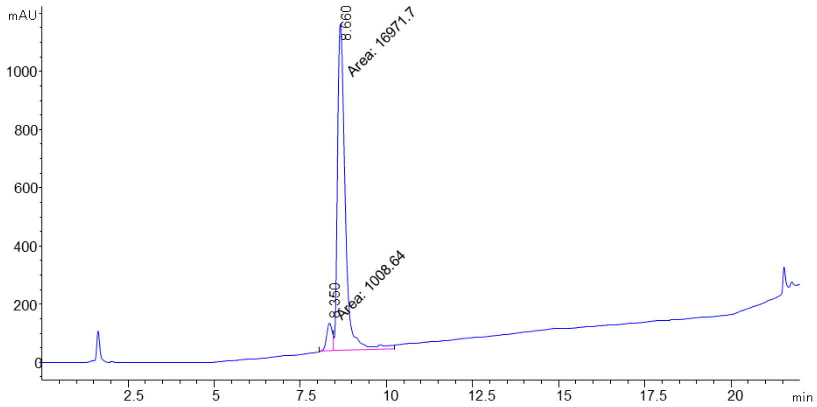

Figure S27: Analytical HPLC chromatogram of purified XG5, r_t = 8.660 min.

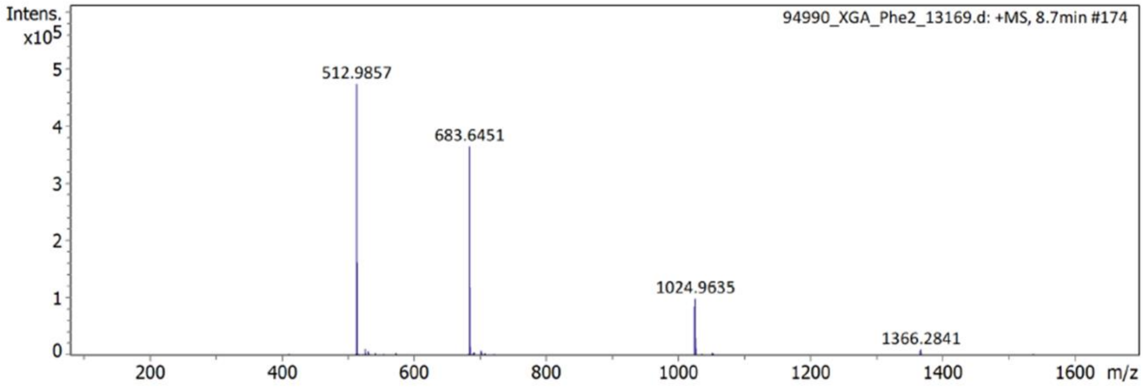

Figure S28: ESI-HRMS of purified XG5.

^{nat}In-XG1

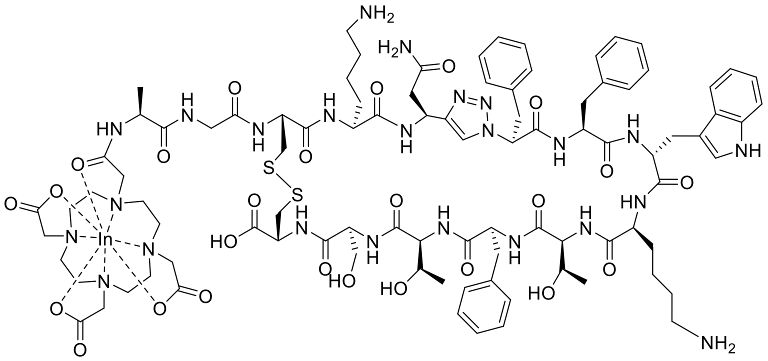

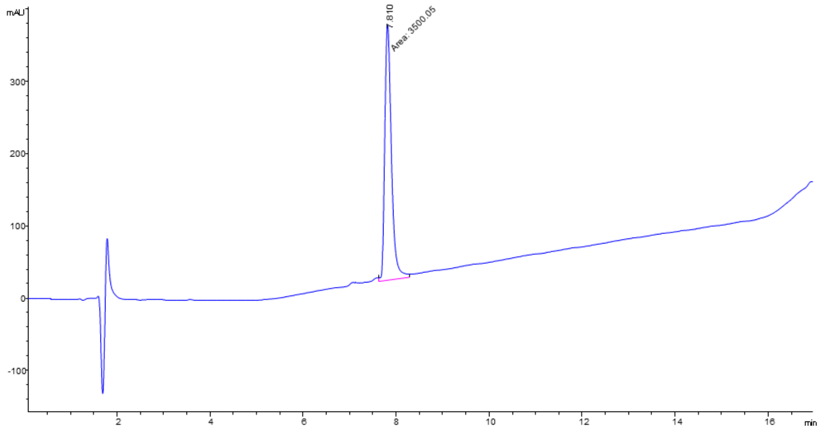

Figure S29: Analytical HPLC chromatogram of purified [^{nat}In]In-XG1, r_t = 7.810 min.

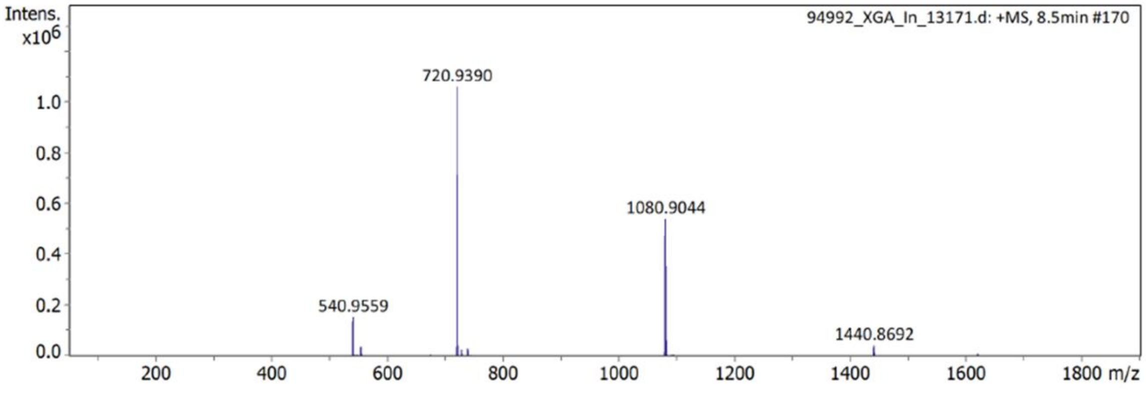

Figure S30: ESI-HRMS of purified [^{nat}In]In-XG1.

TATE ([Tyr³]octreotate)

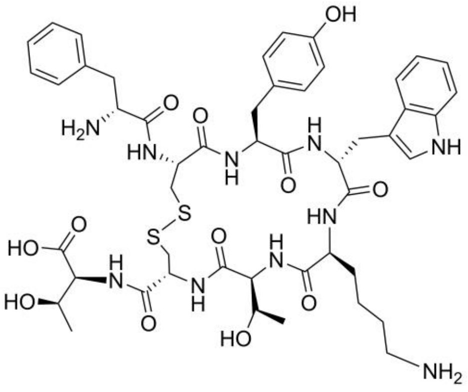

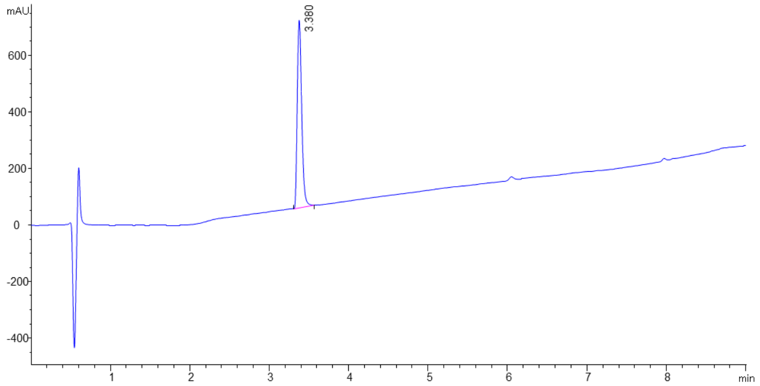

Figure S31: Analytical HPLC chromatogram of purified TATE, r_t = 7.810 min.

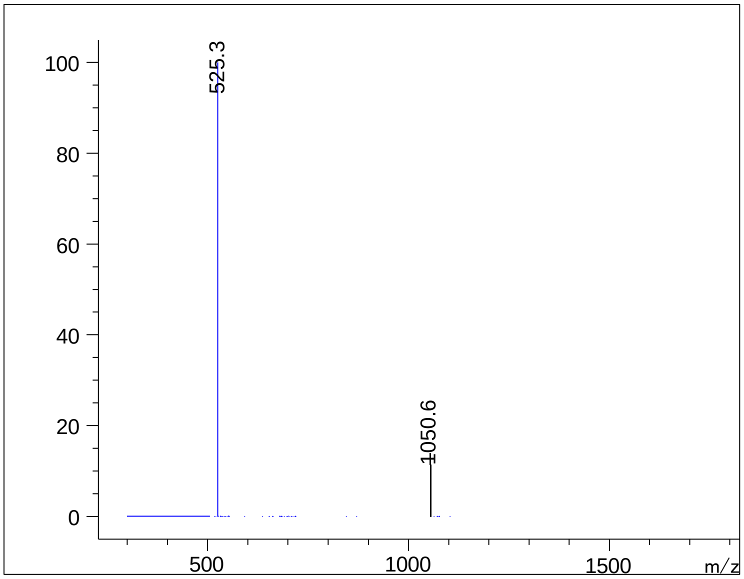

Figure S32: ESI-MS of purified TATE.

6. γ-HPLC chromatograms

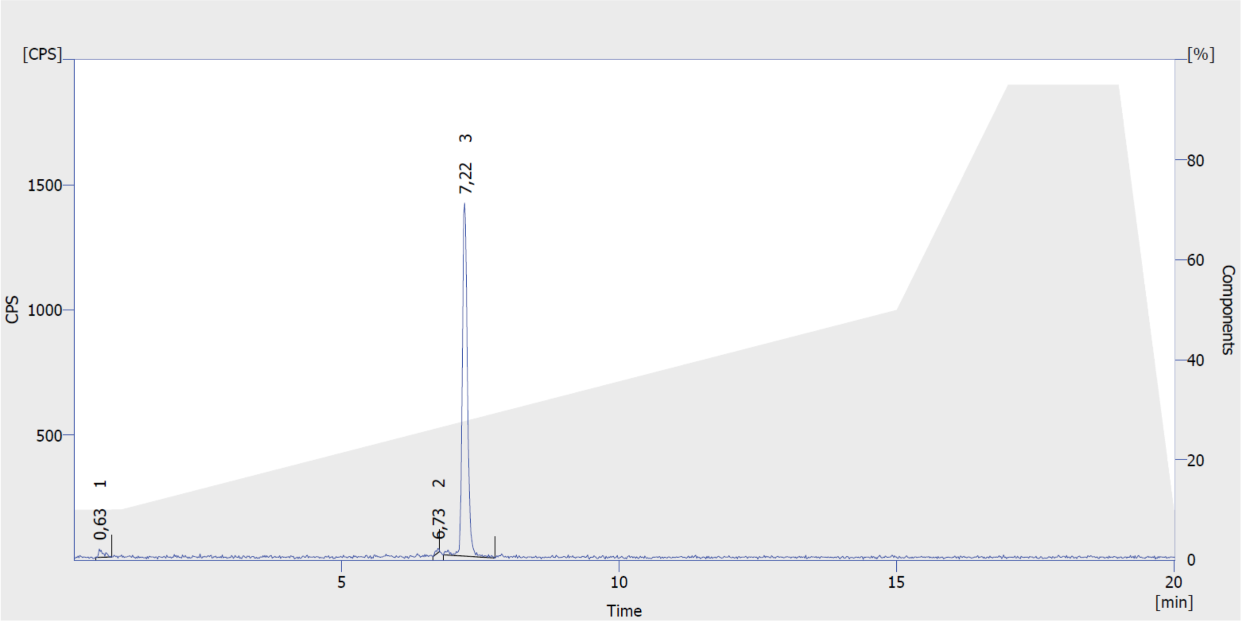

Figure S33: γ-HPLC of [^{111}In]In-AT2S, r_t = 7.22 min.

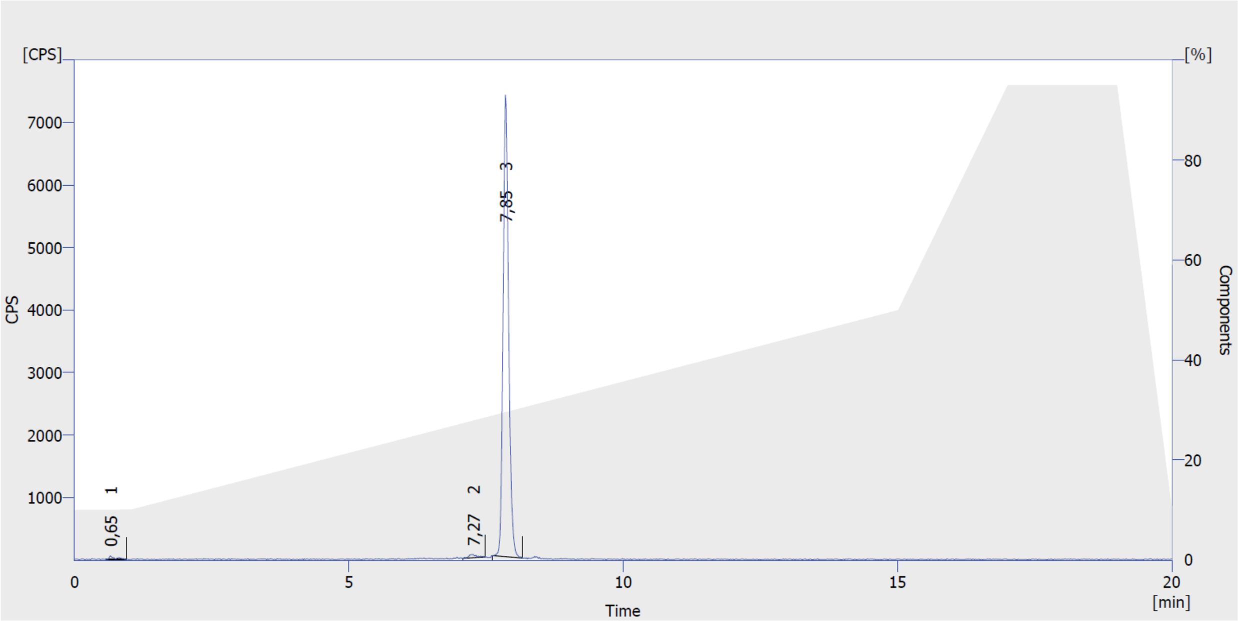

Figure S34: γ-HPLC of [^{111}In]In-XG1, r_t = 7.85 min.

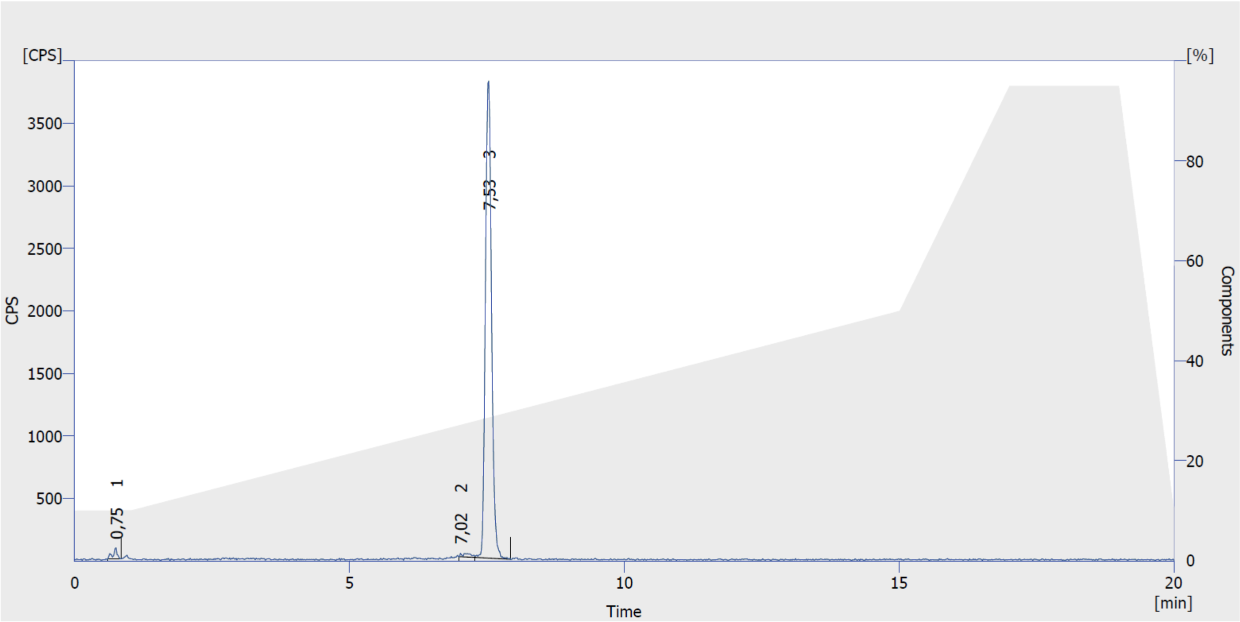

Figure S35: γ-HPLC of [^{111}In]In-XG2, r_t = 7.53 min.

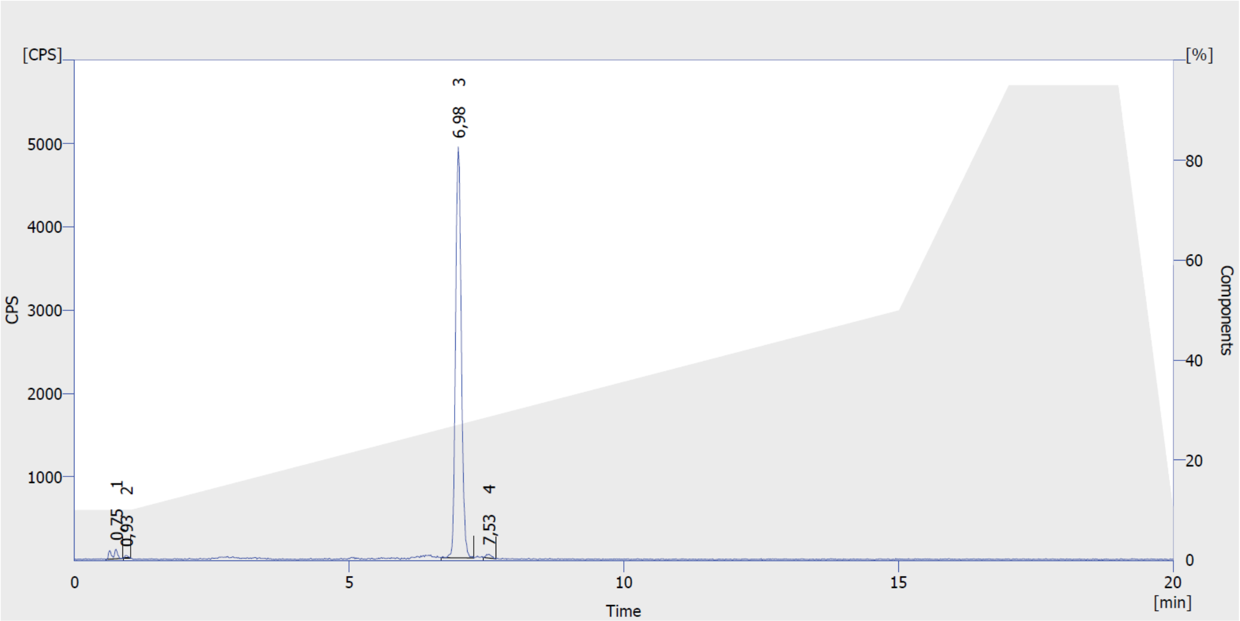

Figure S36: γ-HPLC of [^{111}In]In-XG3, r_t = 6.98 min.

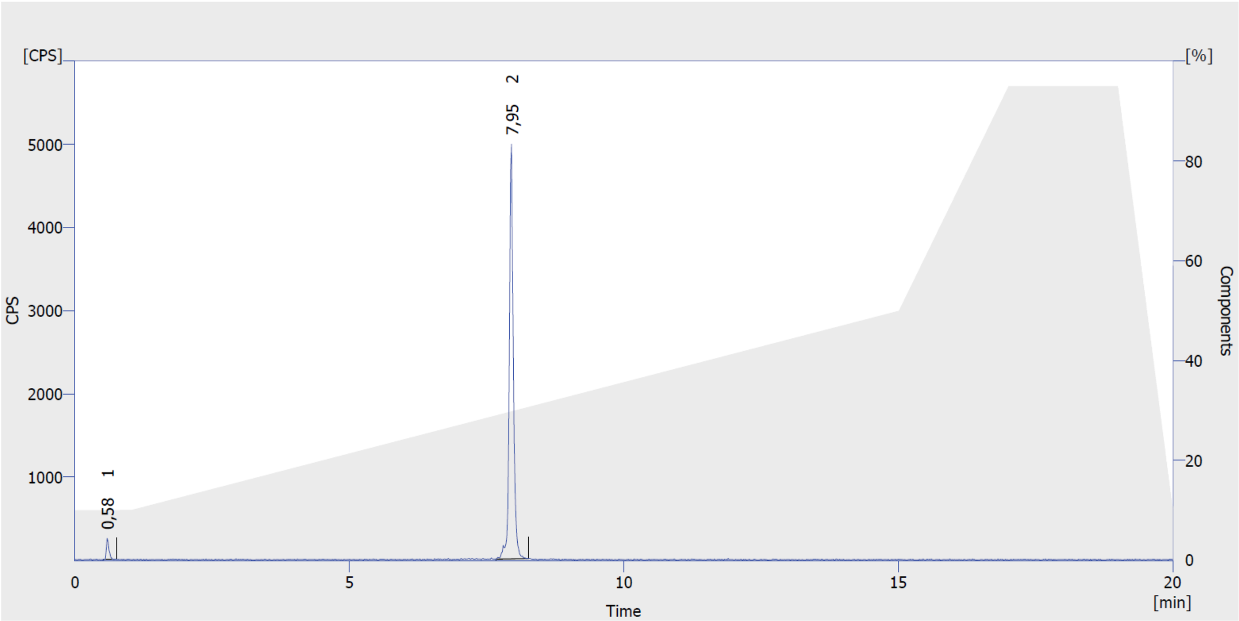

Figure S37: γ-HPLC of [^{111}In]In-XG4, r_t = 7.95 min.

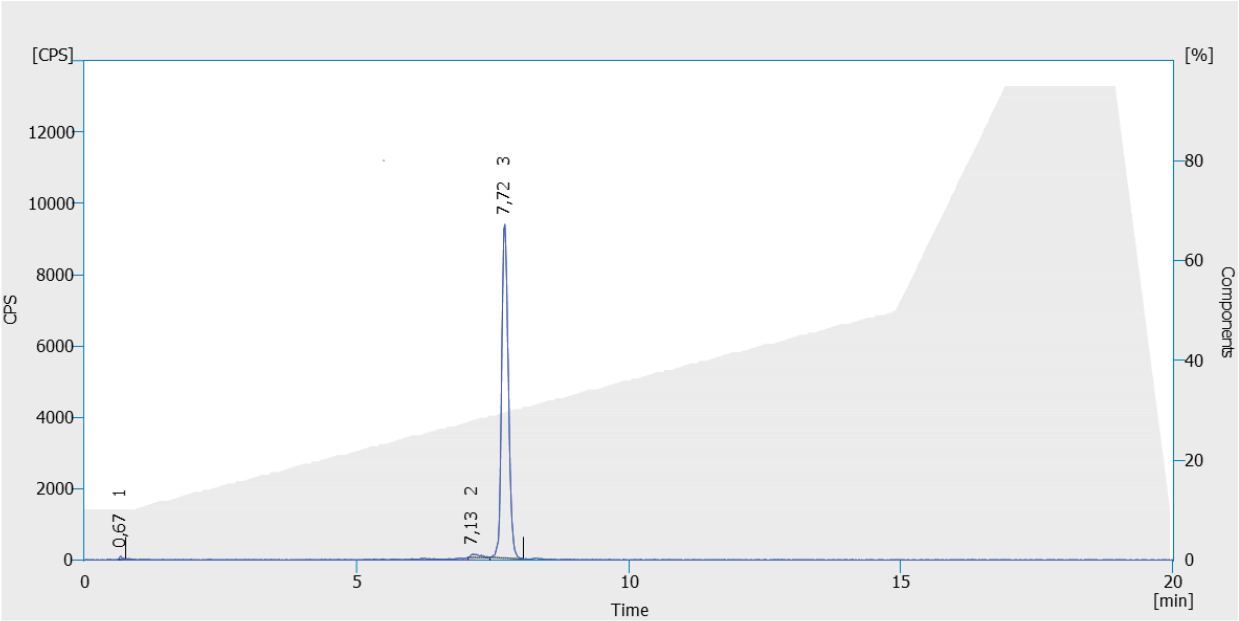

Figure S38: γ-HPLC of [^{111}In]In-XG5, r_t = 7.72 min.

7. Binding and internalizations studies

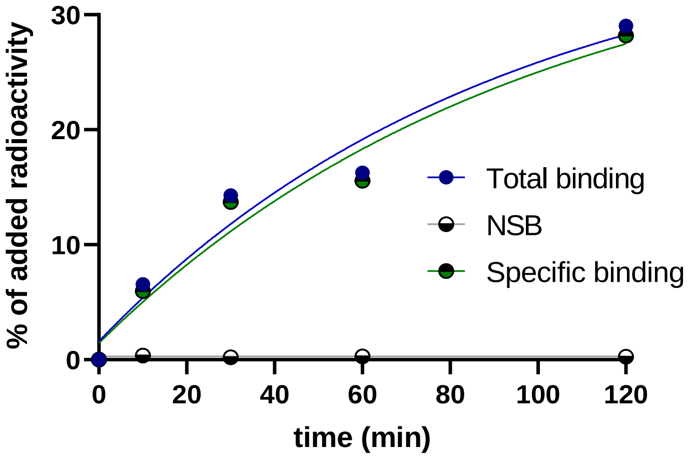

Figure S39: Binding studies of [^{111}In]In-XG1 in AR42J cell line. NSB stands for non-specific binding.

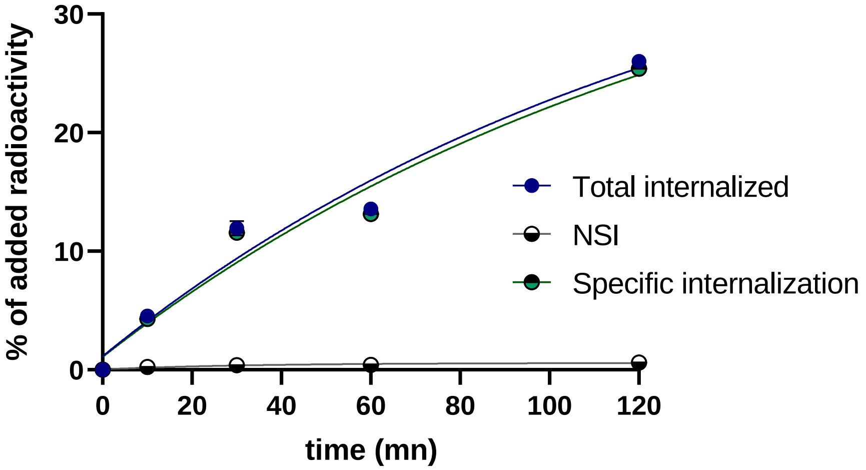

Figure S40: Internalization studies of [^{111}In]In-XG1 in AR42J cell line. NSI stands for non-specific internalization.

8. Competition binding assays

hSST$_1$R

LTT-SST-28: IC$_{50}$ = 0.67 ± 0.26 nM (3)

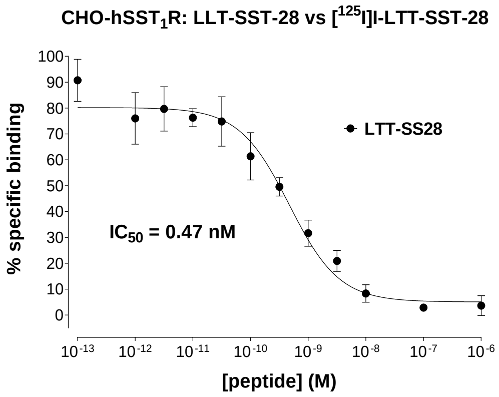

Figure S41: Validation of CHO-hSST$_1$R cell membranes with the LTT-SST-28 pansomatostatin reference.

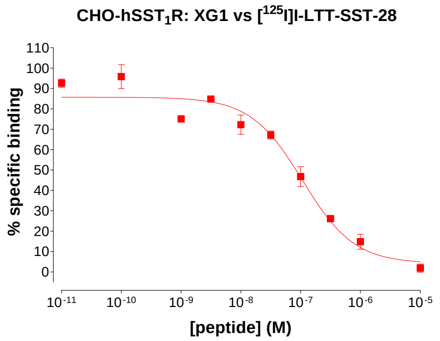

Figure S42: Representative competition binding experiment of XG1 (non-metal tagged) vs [^{125}I]I-LTT-SST-28 in CHO-hSST$_1$R cell membranes.

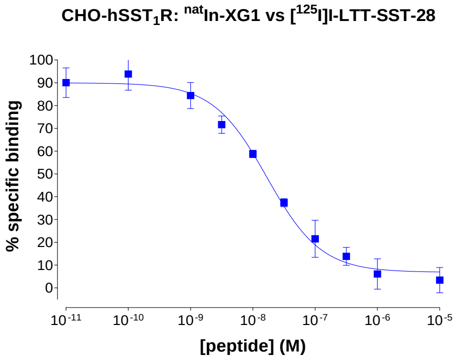

Figure S43: Representative competition binding experiment of ^{nat}In-XG1 vs [^{125}I]I-LTT-SST-28 in CHO-hSST$_1$R cell membranes.

hSST2R

LTT-SST-28: IC50 = 0.33 nM

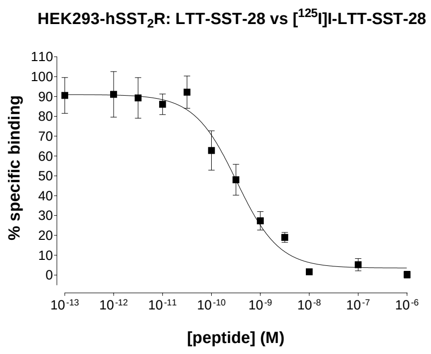

Figure S44: Validation of HEK293-hSST2R cell membranes with the LTT-SST-28 pansomatostatin reference.

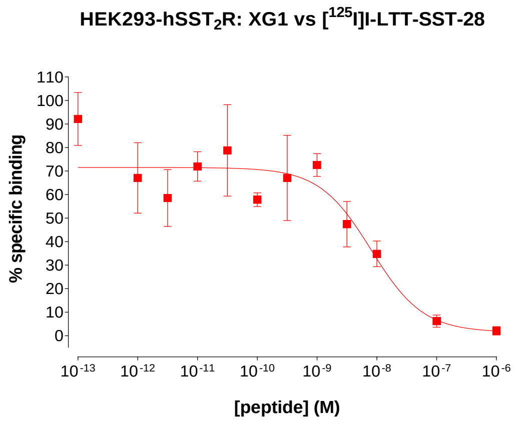

Figure S45: Representative competition binding experiment of XG1 (non-metal tagged) vs [^{125}I]I-LTT-SST-28 in HEK293-hSST2R cell membranes**.**

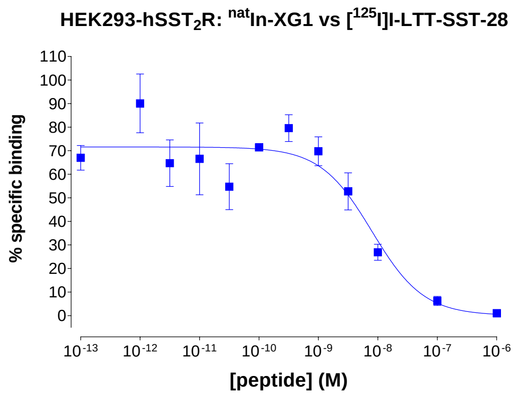

Figure S46: Representative competition binding experiment of ^{nat}In-XG1 vs [^{125}I]I-LTT-SST-28 in HEK293-hSST2R cell membranes.

hSST3R

LTT-SST-28: IC50 = 0.08 nM

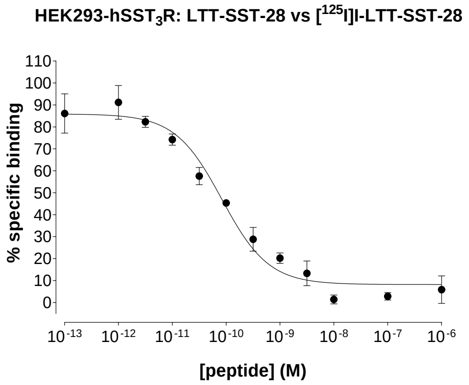

Figure S47: Validation of HEK293-hSST3R cell membranes with the LTT-SST-28 pansomatostatin reference.

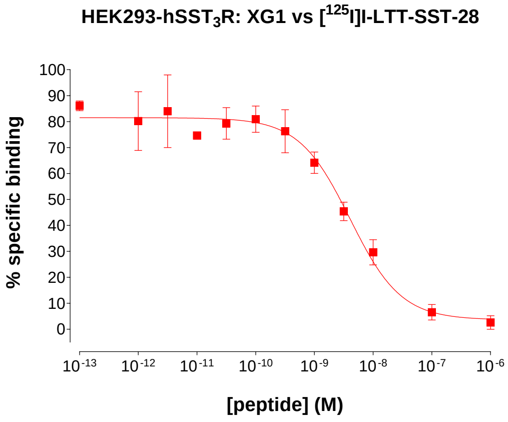

Figure S48: Representative competition binding experiment of XG1 (non-metal tagged) vs [^{125}I]I-LTT-SST-28 in HEK293-hSST3R cell membranes.

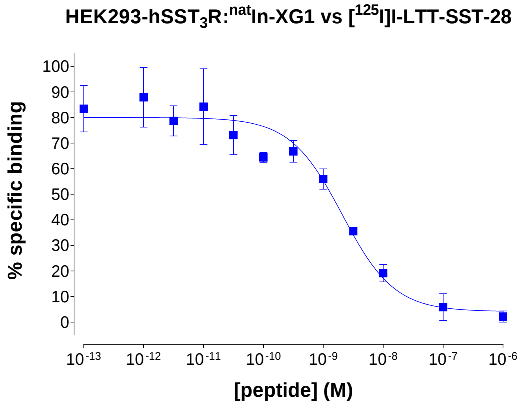

Figure S49: Representative competition binding experiment of ^{nat}In-XG1 vs [^{125}I]I-LTT-SST-28 in HEK293-hSST3R cell membranes.

hSST5R

LTT-SST-28: IC_{50} = 0.11 nM

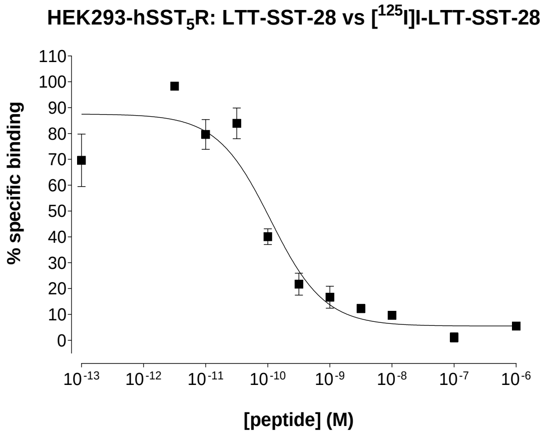

Figure S50: Validation of HEK293-hSST5R cell membranes with the LTT-SST-28 pansomatostatin reference.

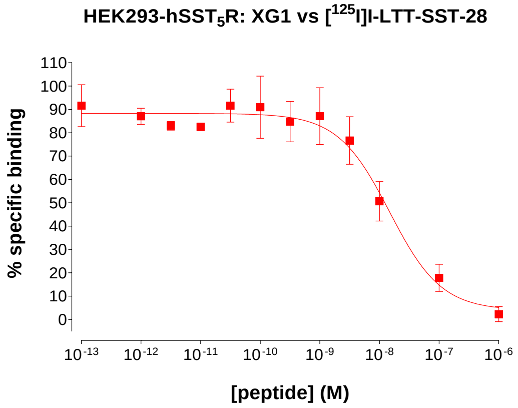

Figure S51: Representative competition binding experiment of XG1 (non-metal tagged) vs [^{125}I]I-LTT-SST-28 in HEK293-hSST5R cell membranes.

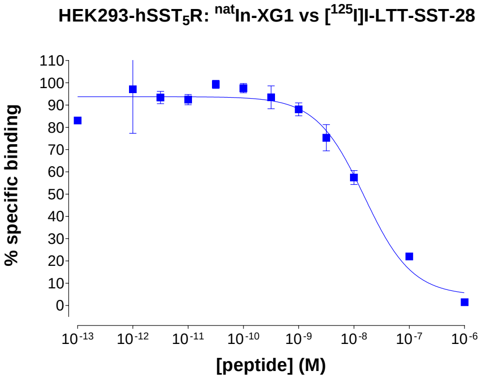

Figure S52: Representative competition binding experiment of ^{nat}In-XG1 vs [^{125}I]I-LTT-SST-28 in HEK293-hSST5R cell membranes.

9. Structure of Entresto® and *in vivo* release of sacubitrilat

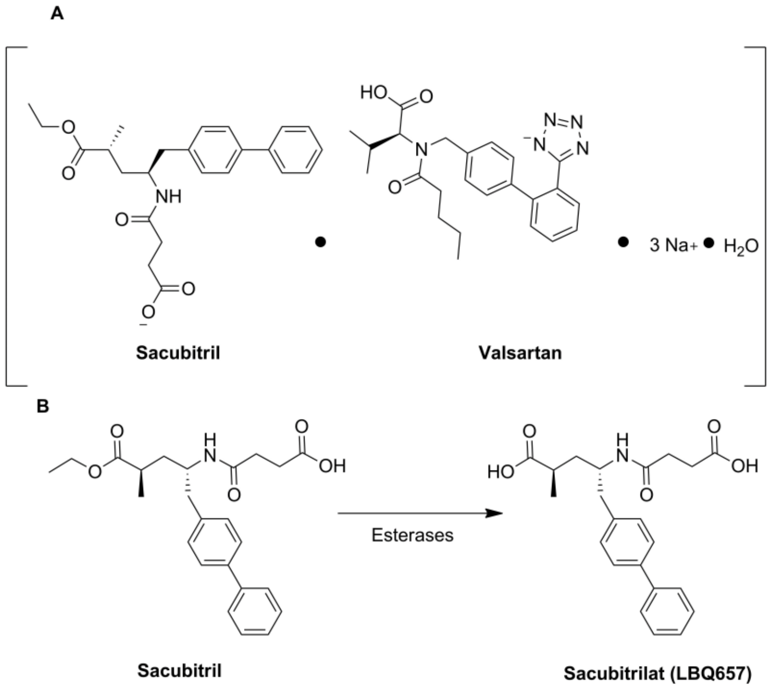

Figure S53: (a) Structure of the molecules contained in Entresto®, Sacubitril and Valsartan and (**b**) Release of sacubitrilat by esterases (LBQ657)[4].

10. Metabolic stability experiments

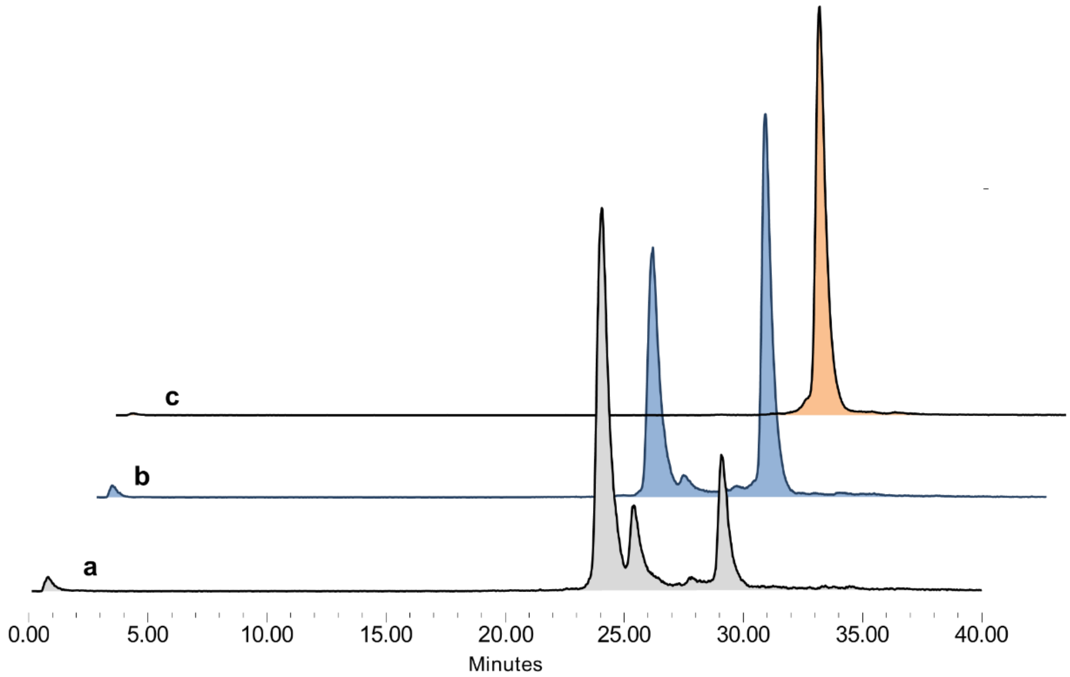

Figure S54: γ-HPLC chromatogram of the metabolic stability (**a**) of [^{111}In]In-XG1 (grey), (**b**) by co-injection of the labeling solution reference with the blood sample on the column (blue) and (**c**) quality control of [^{111}In]In-XG1 (red**).**

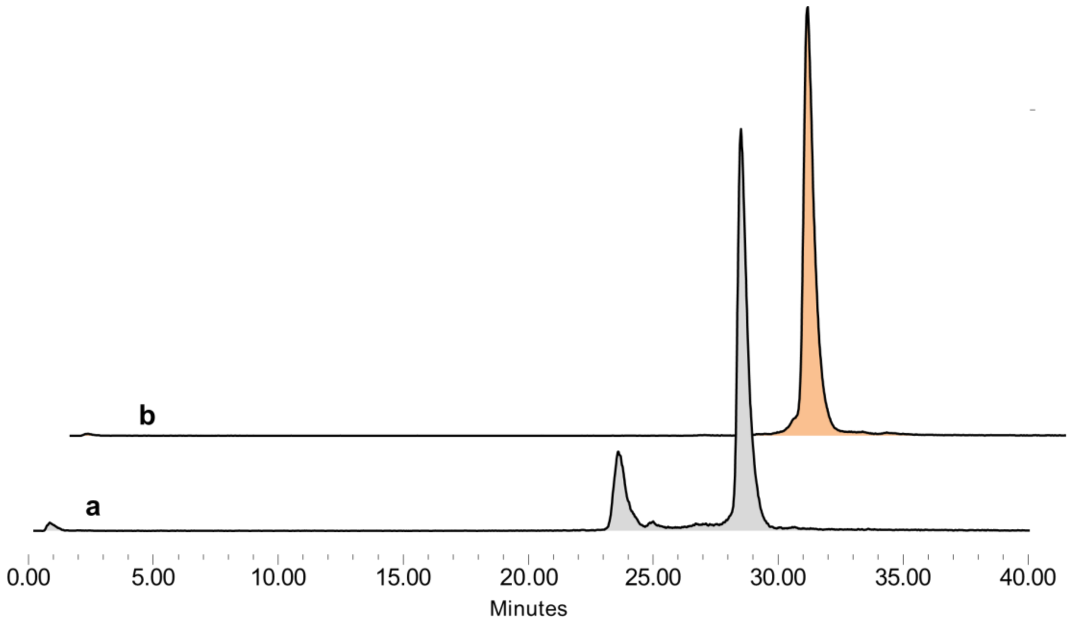

Figure S55: γ-HPLC chromatogram of the metabolic stability of (**a**) [^{111}In]In-XG1 in the Entresto® treated mice (grey) and (**b**) quality control of [^{111}In]In-XG1 (red).

11. Tumor/kidney uptake studies

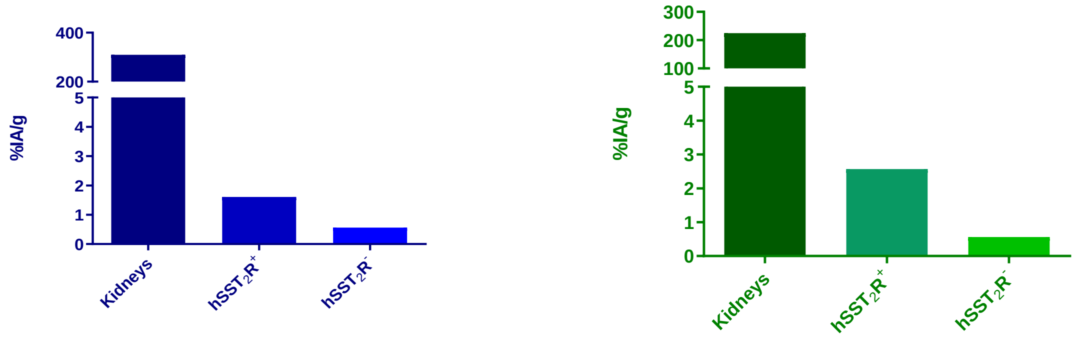

(**a**) (**b**)

Figure S56: Estimation of radioactivity uptake of $[^{111}In]$In-XG1 at 4 h pi (**a**) selectively shown for kidneys (310%IA/g), HEK293-hSST$_2$R tumor (1.61%IA/g) and wtHEK293 tumor (0.56%IA/g) in a control mouse (blue bars) and (**b**) kidneys (225%IA/g), HEK293-hSST$_2$R (2.57%IA/g) and wtHEK293 (0.56%IA/g) in a mouse pretreated with Entresto® (green bars).

12. References

1 Grob, N. M.; Häussinger, D.; Deupi, X.; Schibli, R.; Behe, M.; Mindt, T. L. Triazolo-Peptidomimetics: Novel Radiolabeled Minigastrin Analogs for Improved Tumor Targeting. *J Med Chem* **2020**, *63* (9), 4484–4495. https://doi.org/10.1021/acs.jmedchem.9b01936.

2 Wünsch, M.; Schröder, D.; Fröhr, T.; Teichmann, L.; Hedwig, S.; Janson, N.; Belu, C.; Simon, J.; Heidemeyer, S.; Holtkamp, P.; Rudlof, J.; Klemme, L.; Hinzmann, A.; Neumann, B.; Stammler, H. G.; Sewald, N. Asymmetric Synthesis of Propargylamines as Amino Acid Surrogates in Peptidomimetics. *Beilstein Journal of Organic Chemistry* **2017**, *13*, 2428–2441. https://doi.org/10.3762/bjoc.13.240.

3 Tatsi, A.; Maina, T.; Cescato, R.; Waser, B.; Krenning, E. P.; de Jong, M.; Cordopatis, P.; Reubi, J. C.; Nock, B. A. [^{111}In-DOTA]Somatostatin-14 Analogs as Potential Pansomatostatin-like Radiotracers - First Results of a Preclinical Study. *EJNMMI Res* **2012**, *2* (1), 1. https://doi.org/10.1186/2191-219X-2-25.

4 Ohisi, M. Sacubitril/Valsartan-A new weapon for fighting the hypertension paradox. *Hypertens. Res* **2022**, 45, 915–916. https://doi.org/10.1038/s41440-022-00872-w.
